# Scaffold hopping and optimisation of 3’,4’-dihydroxyphenyl- containing thienopyrimidinones: synthesis of quinazolinone derivatives as novel allosteric inhibitors of HIV-1 reverse transcriptase-associated ribonuclease H

**DOI:** 10.1080/14756366.2020.1835884

**Published:** 2020-11-03

**Authors:** Graziella Tocco, Francesca Esposito, Pierluigi Caboni, Antonio Laus, John A. Beutler, Jennifer A. Wilson, Angela Corona, Stuart F. J. Le Grice, Enzo Tramontano

**Affiliations:** a Department of Life and Environmental Sciences, University of Cagliari, Cittadella Universitaria di Monserrato, Cagliari, Italy; b Molecular Targets Program, National Cancer Institute, Frederick, MD, USA; c Basic Research Laboratory, National Cancer Institute, Frederick, MD, USA

**Keywords:** Bioisosters, RNase H allosteric inhibitors, HIV-1 virus, RNase H, integrase

## Abstract

Bioisosteric replacement and scaffold hopping are powerful strategies in drug design useful for rationally modifying a hit compound towards novel lead therapeutic agents. Recently, we reported a series of thienopyrimidinones that compromise dynamics at the p66/p51 HIV-1 reverse transcriptase (RT)-associated Ribonuclease H (RNase H) dimer interface, thereby allosterically interrupting catalysis by altering the active site geometry. Although they exhibited good submicromolar activity, the isosteric replacement of the thiophene ring, a potential toxicophore, is warranted. Thus, in this article, the most active 2-(3,4-dihydroxyphenyl)-5,6-dimethylthieno[2,3-d]pyrimidin-4(3*H*)-one **1** was selected as the hit scaffold and several isosteric substitutions of the thiophene ring were performed. A novel series of highly active RNase H allosteric quinazolinone inhibitors was thus obtained. To determine their target selectivity, they were tested against RT-associated RNA-dependent DNA polymerase (RDDP) and integrase (IN). Interestingly, none of the compounds were particularly active on (RDDP) but many displayed micromolar to submicromolar activity against IN.

## Introduction

Since the beginning of the AIDS epidemic, almost 80 million people have been infected with human immunodeficiency virus, type 1 (HIV-1), and currently an estimated 38 million people are infected worldwide^1^. Unfortunately, a treatment that eradicates the virus from infected people^2^ or a vaccine^3^ is still not available. At present, the challenge of a successful antiretroviral therapy is to convert a terminal disease into a manageable chronic infection by reducing HIV-1 levels in the blood (viral load). In this regard, the advent of the combination active antiretroviral therapy (cART) represents a major achievement in reducing mortality and morbidity of HIV-1 infected patients^4^. Nowadays, although 27 FDA-approved drugs are available for the treatment of AIDS^5^, antiviral drug discovery has not waned. In fact, the selection and spread of HIV-1 variants resistant to current therapies represent the major clinical problem in the fight against AIDS^6–8^, justifying the increasing demand for new drugs to reinforce the cART arsenal. For that reason, the design of new molecules that target key enzymes of the viral lifecycle with an innovative mode of action could have a tremendous impact on the fight against HIV/AIDS. Reverse transcriptase (RT) together with integrase (IN) and protease (PR), is one of the three viral enzymes essential for HIV-1 replication. DNA polymerase and Ribonuclease H (RNase H) are the two enzymatic activities of RT which catalyse conversion of single-stranded viral RNA into integration-competent, double-stranded linear DNA. Many site-directed mutagenesis studies have demonstrated the essential role of the RNase H domain in virus infectivity, also indicating that the retrovirus-associated activity is unlikely to be supplemented by a host enzyme^9^^,^^10^. This observation suggests RT-associated RNase H as an attractive target that may open opportunities for new systematic HIV-drug discovery efforts^11–15^. In fact, several RNase H inhibitors have been developed over the last two decades, with promising results against drug-resistant variants^16–20^ the majority of which belonging to the class of active site inhibitors^21^. Unfortunately, many representatives of this class can be easily displaced by the nucleic acid substrate^22^ and this drawback has made the progression of RNase H active site inhibitors towards clinical trials challenging. Consequently, attention moved to a less populated class of compounds able to allosterically tackle the viral RNase H function. Such an approach appeared extremely advantageous since the inhibition of related host enzymes, especially those of the polynucleotidyl phosphotransferase class, might be avoided. In this regard, we have recently reported a series of differently decorated thienopyrimidinones, that compromise dynamics at the p66/p51 HIV-1 RT-RNase H dimer interface and consequently interrupting catalysis by altering the active site geometry.^11^

Interestingly, all these compounds, in contrast to active site RNase H inhibitors, were able to destabilise HIV-1 RT both in the absence and presence of the nucleic acid substrate, decreasing the melting temperature of the enzyme in some cases by more than 5 °C. In addition, by using a panel of drug-resistant and -sensitive recombinant enzymes we could identify several thienopyrimidinone derivatives which retained activity against drug-resistant variants and 2–(3,4-dihydroxyphenyl)-5,6-dimethylthieno[2,3-d]pyrimidin-4(3*H*)-one **1** emerged as the best compound (wt RT RNase H IC_50_ = 0.26 μM and C280A RT RNase H IC_50_ = 0.32 μM)^12^.

**Figure 1.**
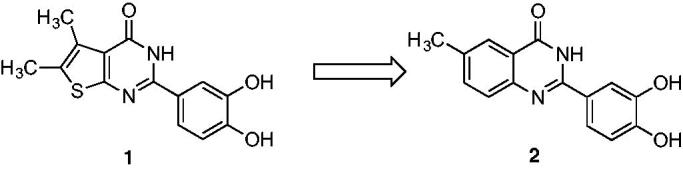
Bioisosteric core replacement.

Since bioisosterism is a well-known strategy for rational lead optimisation in drug discovery^23^ particularly to improve pharmacological activity, we applied it in an effort to enhance the thienopyrimidinone **1** biological effects. The initial candidate, 2–(3,4-dihydroxyphenyl)-6-methylquinazolin-4(3*H*)-one **2,** showed promising activities, inhibiting HIV-1 RNase H in a submicromolar range (IC_50_ = 0.41 µM) (Figure 1 and Table 1). From a structural perspective, our studies indicated that 3,4-dihydroxyphenyl moiety was crucial for activity of every inhibitor, while the bioisosteric substitution of the thiophene core with a 6-methyl phenyl unit as in compound **2** slightly affected the activity^12^ (Figure 1). In fact, since the thiophene ring is a potential toxicophore^24^, with a strict tropism for liver, kidneys, and immune system^25^, its isosteric replacement in biologically active molecules is desirable.

**Table 1.** Inhibition of wild type and p66/p51C280A mutant HIV-1 RNase H activity by compounds **2**–**17**. 
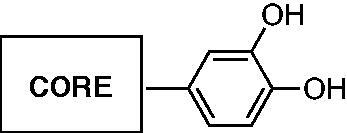

Compound	CORE	IC_50_ (µM)	
		WT	C280A
**2**^12^		0.41 ± 0.01	0.37 ± 0.01
**3**		0.50 ± 0.02	0.23 ± 0.02
**4**		0.57 ± 0.02	0.41 ± 0.01
**5**		0.73 ± 0.02	0.33 ± 0.003
**6**		0.37 ± 0.01	0.29 ± 0.006
**7**		1.1 ± 0.04	0.51 ± 0.05
**8**		0.15 ± 0.01	0.12 ± 0.001
**9**		0.45 ± 0.008	0.40 ± 0.01
**10**		0.31 ± 0.01	0.20 ± 0.01
**11**		0.43 ± 0.01	0.66 ± 0.05
**12**		0.52 ± 0.06	0.84 ± 0.03
**13**		0.31 ± 0.01	0.17 ± 0.004
**14**		0.56 ± 0.25	0.40 ± 0.03
**15**		0.64 ± 0.03	0.39 ± 0.01
**16**		2.4 ± 1.6	2.0 ± 0.1
**17**^27^		3.46 ± 0.47	–

Similar to thienopyrimidinones, we speculated that decorations of the benzene ring might positively affect inhibitory activity since the recently reported^26^ 2–(3,4-dihydroxyphenyl)-quinazolin-4(3*H*)-one **(17)** is an 8-fold less potent HIV-1 RNase H inhibitor. Based on this evidence, we elected to investigate the introduction of different groups on the phenyl ring in order to establish their inhibitory role. A total of 22 new compounds were synthesised and tested against wt and C280A RT, also investigating their mode of action by means of magnesium-complexation and differential scanning fluorimetry experiments. Furthermore, since many compounds are reported to be dual inhibitors of different HIV-1 functions^27^^,^^28^, to determine target specificity, we tested the effect of the entire set of quinazolinone-based derivatives on RDDP and IN activities. We also tested the compounds in a cell-based antiviral system.

## Material and methods

### Chemistry

All reagents were purchased from commercial sources and used without further purification. TLC performed on Aldrich silica gel 60 F254 (0.25 mm) with detection by UV was used for reaction monitoring. ^1^H and ^13 ^C NMR spectra were recorded on a Varian Unity Inova 500 MHz spectrometer. High-resolution mass spectra were recorded using an Agilent 6520 LC-QTOF-MS system.

### General procedure for arylpyrimidinone core formation

A mixture of the appropriate 2-amino arylamide (1.14 mmol), molecular iodine (1.48 mmol), and the appropriate aromatic aldehyde (1.37 mmol) in the presence of acetonitrile as solvent (20 ml) was stirred at room temperature for 6 h (TLC). The reaction was quenched with 40 ml of 5% Na_2_S_2_O_3_ solution, and the resulting precipitate was filtered off. The crude product was washed with *n*-hexane/ethyl acetate (1:1) and recrystallised from ethanol to give the final arylpyrimidinone as pure product.

For compounds **4** and **5**, the corresponding 2-amino arylamide was prepared as follow.

To a magnetically stirred suspension of stannous chloride (24.68 mmol) in concentrated HCl (37%) (9.7 ml), the appropriate 2-nitrobenzamide (8.52 mmol) was added in about 1 h, maintaining the temperature of the slurry below 5 °C. After addition was complete, the mixture was stirred for 30 h at room temperature. A white slurry was obtained which was diluted with cold water (130 ml) and then treated with aqueous NaOH 40% (40 ml) to dissolve the tin salt. The mixture was extracted with ethyl acetate (4 × 100 ml), and the extracts dried on Na_2_SO_4_ and concentrated under *vacuum* to obtain the pure 2-amino arylamide.

*2–(3,4-dihydroxyphenyl)-6-methylquinazolin-4(3H)-one* (**2**). Yield (%) = 78. ^1^H NMR (500 MHz, DMSO): δ 12.12 (s, 1H), 9.59 (bs, 1H), 9.29 (bs, 1H), 7.91 (s, 1H), 7.67(s, 1H), 7.61 (d, *J* = 8.0 Hz, 1H), 7.57–7.52 (dd, *J* = 7.1 Hz, 2H), 6.83 (d, *J* = 8.0 Hz, 1H), 2.44 (s, 3H) ppm. ^13 ^C NMR (500 MHz, DMSO): δ 161.17, 155.29, 148.12, 147.01, 146.76, 145.18, 137.56, 133.71, 128.17, 122.43,120.11, 117.17, 116.15, 21.01 ppm. HRMS calculated for C_15_H_12_N_2_O_3_ 268.08, found 268.07.

*2–(3,4-dihydroxyphenyl)-7-methylquinazolin-4(3H)-one* (**3**). Yield (%) = 68. ^1^H NMR (500 MHz, DMSO): δ 12.06 (s, 1H), 9.11 (bs, 1H), 8.48 (bs, 1H), 7.98 (d, *J* = 7.5 Hz, 1H), 7.66 (s, 1H), 7.53 (d, *J* = 8.0 Hz, 1H), 7.46 (s, 1H), 7.27 (d, *J* = 8.0 Hz, 1H), 6.83 (d, *J* = 7.5 Hz, 1H), 2.45 (s, 3H) ppm. ^13 ^C NMR (500 MHz, DMSO): δ 161.23, 155.45 148.67, 147.11, 146.56, 145.01, 137.58, 133.88, 128.16, 122.42,120.01, 117.00, 116.15, 20.56 ppm. HRMS calculated for C_15_H_12_N_2_O_3_ 268.08, found 268.09.

*2–(3,4-dihydroxyphenyl)-6-methoxyquinazolin-4(3H)-one* (**4**). Yield (%) = 73. ^1^H NMR (500 MHz, DMSO): δ 12.16 (s, 1H), 9.56 (bs, 1H), 9.23 (bs, 1H), 7.65 (s, 1H), 7.61 (d, *J* = 8.5 Hz, 1H), 7.51 (d, *J* = 6.0 Hz, 1H), 7.40 (d, *J* = 8.5 Hz, 1H), 6.82 (d, *J* = 6.0 Hz, 1H), 3.88 (s, 3H) ppm. ^13^C NMR (500 MHz, DMSO): δ 162.11, 153.99, 151.14, 148.78, 135.88, 129.00, 122.33, 119.10, 118.36, 116.11, 115.57, 114.38, 57.04 HRMS calculated for C_15_H_12_N_2_O_4_ 284.08, found 284. 10.

*2–(3,4-dihydroxyphenyl)-6,7-dimethoxyquinazolin-4(3H)-one* (**5**). Yield (%) = 74. ^1^H NMR (500 MHz, DMSO): δ 12.07 (s, 1H), 9.60 (bs, 1H), 9.19 (bs, 1H), 7.63 (s, 1H), 7.50 (d, *J* = 7.0 Hz, 1H), 7.43 (s, 1H), 7.10 (s, 1H), 6.81 (d, *J* = 7.0 Hz, 1H), 3.90 (s, 3H), 3.85 (s, 3H) ppm. ^13^C NMR (500 MHz, DMSO): δ 162.21, 154.45, 151.16, 148.12, 135.89, 129.09, 122.23, 119.00, 118.34, 116.78, 115.57, 114.38, 57.08 ppm. HRMS calculated for C_16_H_14_N_2_O_5_ 314.09, found 314.10.

*2–(3,4-dihydroxyphenyl)-6-phenylquinazolin-4(3H)-one* (**6**). Yield (%) = 77. ^1^H NMR (500 MHz, DMSO): δ 12.35 (s, 1H), 9.69 (bs, 1H), 9.29 (bs, 1H), 8.37 (s, 1H), 8.07 (d, *J* = 7.0 Hz, 1H), 7.67–7.56 (m, 3H), 7.45 (s, 1H), 7.37–7.28 (m, 3H), 6.83 (dd, *J* = 6.7 Hz, 2H) ppm. ^13^C NMR (500 MHz, DMSO): δ 165.00, 155.61, 148.23, 141.09,138.12, 135.64, 135.20, 134.88, 133.56, 129.76,128.23, 127.22, 126.88, 124.67, 122.34, 120.54, 118.22, 117.11 ppm. HRMS calculated for C_20_H_14_N_2_O_3_ 330.10, found 330.07.

*2–(3,4-dihydroxyphenyl)benzo[g]quinazolin-4(3H)-one* (**7**). Yield (%) = 80. ^1^H NMR (500 MHz, DMSO): δ 12.02 (s, 1H), 9.52 (bs, 1H), 9.23 (bs, 1H), 8.43 (d, *J* = 7.2 Hz, 2H), 8.23 (d, *J* = 7.5 Hz, 1H), 8.11 (d, *J* = 7.5 Hz, 1H), 7.32 (s, 1H), 7.03 (t, *J* = 7.5 Hz, 1H), 6.98 (t, *J* = 7.5 Hz, 1H), 6.84 (d, *J* = 8.1 Hz, 1H), 6.73 (d, *J* = 8.1 Hz, 1H) ppm. ^13^C NMR (500 MHz, DMSO): δ 160.00, 155.66, 153.09, 150.12, 147.89, 145.43, 140.06, 138.24, 133.56, 132.16, 130.87, 126.11, 122.34, 120.56, 114.98, 111.22 ppm. HRMS calculated for C_18_H_12_N_2_O_3_ 304.08, found 304.07.

*2–(3,4-dihydroxyphenyl)-6-iodoquinazolin-4(3H)-one* (**8**). Yield (%) = 87. ^1^H NMR (500 MHz, DMSO): δ 11.56 (s, 1H), 9.45 (bs, 1H), 9.27 (bs, 1H), 8.35 (s, 1H), 8.03 (d, J = 8.0 Hz, 1H), 7.67(d, J = 8.0 Hz, 1H), 7.13 (s, 1H), 6.83 (d, J = 7.0 Hz, 1H), 6.78 (d, J = 7.0 Hz, 1H) ppm. ^13 ^C NMR (500 MHz, DMSO): δ 162.00, 160.54,154.27, 147.12, 146.04, 145.76,138.59, 134.73, 127.17, 122.22, 119.19, 116.15, 110.90 ppm. HRMS calculated for C_14_H_9_IN_2_O_3_ 379.97, found 379.97.

*2–(3,4-dihydroxyphenyl)-6-bromoquinazolin-4(3H)-one* (**9**). Yield (%) = 79. ^1^H NMR (500 MHz, DMSO): δ 12.11 (s, 1H), 9.56 (bs, 1H), 9.33 (bs, 1H), 8.15 (s, 1H), 7.98 (d, *J* = 8.2 Hz, 1H), 7.87 (d, *J* = 8.2 Hz, 1H), 7.22 (s, 1H), 6.95 (d, *J* = 7.5 Hz, 1H), 6.76 (d, *J* = 7.5 Hz, 1H) ppm. ^13 ^C NMR (500 MHz, DMSO): δ 162.00, 155.21, 148.22, 147.04, 145.15, 144.17, 137.09, 134.07, 125.13, 121.22, 118.00, 116.00, 100.10 ppm. HRMS calculated for C_14_H_9_BrN_2_O_3_ 331.98 found 331.97.

*2–(3,4-dihydroxyphenyl)-6-chloroquinazolin-4(3H)-one* (**10**). Yield (%) = 90. ^1^H NMR (500 MHz, DMSO): δ 12.34 (s, 1H), 9.47 (bs, 1H), 9.38 (bs, 1H), 8.02(s, 1H), 7.78 (d, *J* = 8.0 Hz, 1H), 7.67 (s, 1H), 7.54 (d, *J* = 7.5 Hz, 1H), 6.83 (d, *J* = 8.0 Hz, 1H), 6.79 (d, *J* = 7.5 Hz, 1H) ppm. ^13 ^C NMR (500 MHz, DMSO): δ 162.34, 156.27, 151.01, 149.04, 147.66, 145.89, 137.12, 135.77, 123.00, 121.45, 118.50, 111.76 ppm. HRMS calculated for C_14_H_9_ClN_2_O_3_ 288.03 found 288.02.

*2–(3,4-dihydroxyphenyl)-7-chloroquinazolin-4(3H)-one* (**11**). Yield (%) = 87. ^1^H NMR (500 MHz, DMSO): δ 12.45 (s, 1H), 9.82 (bs, 1H), 9.42 (bs, 1H), 8.20 (d, *J* = 10 Hz, 1H), 7.79 (d, *J* = 8.9 Hz, 1H), 7.77 (s, 1H), 7.67 (d, *J* = 10 Hz, 1H), 7.60 (d, *J* = 8.9 Hz, 1H), 6.95 (d, *J* = 8.9 Hz, 1H) ppm. ^13 ^C NMR (500 MHz, DMSO): δ 162.31, 156.12, 150.99, 149.54, 147.16, 145.87, 137.22, 135.58, 123.16, 121.75, 118.50, 110.60 ppm. HRMS calculated for C_14_H_9_ClN_2_O_3_ 288.03 found 288.03.

*2–(3,4-dihydroxyphenyl)-6-fluoroquinazolin-4(3H)-one* (**12**). Yield (%) = 93. ^1^H NMR (500 MHz, DMSO): δ 12.46 (s, 1H), 9.76 (bs, 1H), 9.39 (bs, 1H), 7.88 (d, *J* = 9.0 Hz, 1H), 7.85 (d, *J* = 8.0 Hz, 1H), 7.79 (s, 1H), 7.77 (d, *J* = 9.0 Hz, 1H), 7.66 (d, *J* = 8.0 Hz, 1H), 6.94 (d, *J* = 8.0 Hz, 1H) ppm. ^13 ^C NMR (500 MHz, DMSO): δ 160.11, 157.22, 151.39, 149.67, 147.28, 145.88, 137.02, 136.56, 123.12, 121.79, 119.00, 111.00 ppm. HRMS calculated for C_14_H_9_FN_2_O_3_ 272.06 found 272.05.

*2–(3,4-dihydroxyphenyl)-6-nitroquinazolin-4(3H)-one* (**13**). Yield (%) = 84. ^1^H NMR (500 MHz, DMSO): δ 12.66 (s, 1H), 9.49 (bs, 1H), 8.78 (bs, 1H), 8.42 (d, *J* = 7.5 Hz, 1H), 8.09 (d, *J* = 8.0 Hz, 1H), 7.80 (s, 1H), 7.78 (d, *J* = 7.5 Hz, 1H), 7.64 (d, *J* = 8.0 Hz, 1H), 6.86 (d, *J* = 7.5 Hz, 1H) ppm. ^13 ^C NMR (500 MHz, DMSO): δ 162.01, 155.12, 151.67, 150.53, 148.12, 146.88, 139.14, 136.58, 123.00, 121.00, 118.10, 115.00 ppm. HRMS calculated for C_14_H_9_N_3_O_5_ 299.05 found 299.05.

*2–(3,4-dihydroxyphenyl)benzofuro[3,2-d]pyrimidin-4(3H)-one* (**15**). Yield (%) = 91. ^1^H NMR (500 MHz, DMSO): δ 12.73 (s, 1H), 9.65 (bs, 1H), 9.31 (bs, 1H), 8.08 (d, *J* = 8.2 Hz, 1H), 7.82 (d, *J* = 7.5 Hz, 1H), 7.69–7.65 (m, 2H), 7.52 (s, 1H), 6.96 (d, *J* = 7.5 Hz, 1H), 6.85 (d, *J* = 8.2 Hz, 1H) ppm. ^13 ^C NMR (500 MHz, DMSO): δ 162.26, 149.89, 146.12, 135.86, 133.22, 123.36, 122.12, 121.09, 120.14, 118.90, 118.21, 113.45 ppm. HRMS calculated for C_16_H_10_N_2_O_4_ 294.06 found 294.05.

*2–(3,4-dihydroxyphenyl)pyrido[2,3-d]pyrimidin-4(3H)-one* (**16**). Yield (%) = 81. ^1^H NMR (500 MHz, DMSO): δ 12.45 (s, 1H), 9.57 (bs, 1H), 9.01 (bs, 1H), 8.77 (d, *J* = 7.8 Hz, 1H), 8.32 (d, *J* = 7.8 Hz, 1H), 7.70 (s, 1H), 7.58 (t, *J* = 7.8 Hz, 1H), 7.12 (d, *J* = 8.0 Hz, 1H), 6.86 (d, *J* = 8.0 Hz, 1H) ppm. ^13 ^C NMR (500 MHz, DMSO): δ 160.91, 159.14, 155.87, 146.22, 139.88, 138.15, 135.76, 133.33, 122.10, 120.00, 117.45, 114.10 ppm. HRMS calculated for C_13_H_9_N_3_O_3_ 255.06 found 255.06.

*2–(3,4-dihydroxyphenyl)quinazolin-4(3H)-one* (**17**). Yield (%) = 93. ^1^H NMR (500 MHz, DMSO): δ 12.20 (s, 1H), 9.63 (bs, 1H), 9.28 (bs, 1H), 8.11 (d, J = 9.0 Hz, 1H), 7.79 (d, J = 9.0 Hz, 1H), 7.67(s, 1H), 7.65 (t, J = 9.0 Hz, 2H), 7.43 (d, J = 8.0 Hz, 1H), 6.85 (d, J = 9.0 Hz, 1H), ppm. ^13 ^C NMR (500 MHz, DMSO): δ 161.00, 159.17, 157.12, 149.72, 145.43, 138.12, 134.96, 133.31, 122.33, 120.00, 116.42, 114.15 ppm. HRMS calculated for C_14_H_10_N_2_O_3_ 254.07 found 254.06.

*2-hydroxy-4-(4-oxo-3,4-dihydroquinazolin-2-yl)benzoic acid* (**18**). Yield (%) = 89. ^1^H NMR (500 MHz, DMSO): δ 13.12 (bs, 1H), 12.15 (s, 1H), 11.06 (bs, 1H), 8.22 (d, *J* = 8.0 Hz, 1H), 7.91 (d, *J* = 8.0 Hz, 1H), 7. 71 (s, 1H), 7.54 (t, *J* = 8.0 Hz, 2H), 7.33 (d, *J* = 7.5 Hz, 1H), 6.98 (d, *J* = 7.5 Hz, 1H), ppm. ^13 ^C NMR (500 MHz, DMSO): δ 172.00, 160.17, 159.24, 151.15, 148.63, 145.12, 139.64, 133.96, 133.11, 121.33, 119.00, 116.22, 111.78 ppm. HRMS calculated for C_15_H_10_N_2_O_4_ 282.06 found 282.04.

*2-(4-amino-3-hydroxyphenyl)quinazolin-4(3H)-one* (**19**). Yield (%) = 83. ^1^H NMR (500 MHz, DMSO): δ 12.34 (s, 1H), 10.03 (bs, 1H), 8.03 (d, *J* = 7.5 Hz, 1H), 7.67 (d, *J* = 7.5 Hz, 1H), 7.54 (s, 1H), 7.11 (t, *J* = 7.5 Hz, 1H), 7.02 (t, *J* = 7.5 Hz, 1H), 6.81 (d, *J* = 8.0 Hz, 1H), 6.76 (d, *J* = 8.0 Hz, 1H), 5.90 (bs, 2H) ppm. ^13 ^C NMR (500 MHz, DMSO): δ 159.20, 157.19, 155.32, 147.79, 144.67, 141.12, 135.00, 122.33, 121.14, 118.65, 116.22, 113.17 ppm. HRMS calculated for C_14_H_11_N_3_O_2_ 253.09 found 253.10.

*2–(3,4-diaminophenyl)quinazolin-4(3H)-one* (**20**). Yield (%) = 82. ^1^H NMR (500 MHz, DMSO): δ 11.98 (s, 1H) 8.25 (d, *J* = 8.0 Hz, 1H), 8.09 (d, *J* = 8.0 Hz, 1H), 7.68 (s, 1H), 7.43 (t, *J* = 8.0 Hz, 2H), 7.04 (d, *J* = 7.4 Hz, 1H), 6.76 (d, *J* = 7.4 Hz, 1H), 5.45 (bs, 2H), 5.34 (bs, 2H) ppm. ^13 ^C NMR (500 MHz, DMSO): δ 163.09, 160.00, 159.12, 152.07, 148.41, 139.12, 136.00, 135.31, 123.13, 121.00, 117.33, 113.18 ppm. HRMS calculated for C_14_H_12_N_4_O 252.10 found 252.09.

*2–(3-hydroxy-4-nitrophenyl)quinazolin-4(3H)-one* (**21**). Yield (%) = 92. ^1^H NMR (500 MHz, DMSO): δ 12.21 (s, 1H), 8.13 (d, *J* = 7.5 Hz, 1H), 7.97 (d, *J* = 7.5 Hz, 1H), 7.34 (s, 1H), 7.15 (t, *J* = 7.5 Hz, 1H), 7.00 (t, *J* = 7.5 Hz, 1H), 6.91 (d, *J* = 8.0 Hz, 1H), 6.80 (d, *J* = 8.0 Hz, 1H), 6.25 (bs, 2H) ppm. ^13 ^C NMR (500 MHz, DMSO): δ 159.70, 156.13, 154.32, 147.43, 145.68, 142.24, 135.10, 126.37, 122.56 117.85, 115.00, 111.17, 110.67 ppm. HRMS calculated for C_14_H_10_N_4_O_3_ 282.08 found 282.08.

### *Synthetic protocols for the preparation of compounds* (14), (22), and (23)

*Synthesis of 6-chloro-2–(3,4-dihydroxyphenyl)-2,3-dihydroquinazolin-4(1H)-one* (**14**). 2-amino-5-chlorobenzamide (0.7 mmol) and 3,4-dihydroxybenzaldehyde (0.91 mmol) were dissolved in a mixture of dichloromethane (10 ml) and acetonitrile (7 ml) and refluxed for 40 h. After completion, the solvent was removed under vacuum. The solid residue was treated with water, filtered and then washed again with water to give compound **14** as a pale yellow crystal. Yield (%) = 82. ^1^H NMR (500 MHz, DMSO): δ 9.42 (s, 1H), 8.47 (bs, 1H), 8.38 (bs, 1H), 7.99 (s, 1H), 7.85 (d, *J* = 7.0 Hz, 1H), 7.54 (d, *J* = 7.5 Hz, 1H), 6.93 (s, 1H), 6.77 (d, *J* = 7.5 Hz, 1H), 6.44 (d, *J* = 7.5 Hz, 1H),6.29 (bs, 1H), 6.13 (s, 1H) ppm. ^13 ^C NMR (500 MHz, DMSO): δ 159.78 149.27, 147.11, 145.64, 137.76, 135.99, 132.00, 124.75, 122.10, 121.00, 116.70, 110.75, 80.05 ppm. HRMS calculated for C_14_H_11_ClN_2_O_3_ 290.05 found 290.05.

*Synthesis of 4-(quinolin-2-yl)benzene-1,2-diol* (**22**). To a mixture of (3,4-dimethoxyphenyl)boronic acid (3.29 mmol), K_2_CO_3_ (3.29 mmol), and Pd(OAc)_2_ (0.027 mmol) in ethanol (9 ml) and water (3 ml) was slowly added 2-bromoquinoline (2.74 mmol). The mixture was stirred at room temperature for 18 h. To the resulting mixture was added water (50 ml), and then extracted with dichloromethane. The organic phase was separated, dried over Mg_2_SO_4_ and concentrated under reduced pressure to give a solid residue which was purified by flash chromatography (silica gel and hexane/acetone 3/1). The pure 2–(3,4-dimethoxyphenyl)quinoline was obtained as light grey solid and used for the next reaction. Yield (%) = 92. ^1^H NMR (500 MHz, DMSO): δ 8.76 (d, *J* = 8.0 Hz, 1H), 8.11 (d, *J* = 7.0 Hz, 1H), 7.93 (d, *J* = 7.0 Hz, 1H), 7.77 (t, *J* = 7.0 Hz, 1H), 7.68 (s, 1H), 7.59 (d, *J* = 7.5 Hz, 1H), 7.55 (t, *J* = 7.0 Hz, 1H),7.29 (d, *J* = 8.0 Hz, 1H), 7.02 (d, *J* = 7.0 Hz, 1H), 3.85 (s, 6H) ppm. ^13 ^C NMR (500 MHz, DMSO): δ 156.00, 153.12, 145.12, 135.82, 130.27, 128.00, 125.26, 122.30, 119.33, 117.01, 115.40, 113.13, 57.89 ppm. HRMS calculated for C_17_H_15_NO_2_ 265.11 found 265.10.

To a stirred solution of 2–(3,4-dimethoxyphenyl)quinoline (0.604 mmol) in dichloromethane (4 ml) at 0 °C was added boron tribromide 1 M solution in dichloromethane (9.66 mmol). After 1.5 h, the resulting mixture was stirred for 4 more hours at room temperature. The resulting mixture was the poured into ice water and the yellow residue obtained was dissolved in ethanol. The mixture was then extracted with dichloromethane and the organic phase, previously dried over anhydrous MgSO_4_, was evaporated to give compound **22** as a yellow solid. Yield (%) = 91. ^1^H NMR (500 MHz, DMSO): δ 9.09 (bs, 1H), 8.87 (bs, 1H), 8.73 (d, *J* = 8.0 Hz, 1H), 8.09 (d, *J* = 7.0 Hz, 1H), 7.89 (d, *J* = 7.0 Hz, 1H), 7.80 (t, *J* = 7.0 Hz, 1H), 7.64 (s, 1H), 7.53 (d, *J* = 7.5 Hz, 1H), 7.51 (t, *J* = 7.0 Hz, 1H),7.29 (d, *J* = 8.0 Hz, 1H), 7.12 (d, *J* = 7.0 Hz, 1H) ppm. ^13 ^C NMR (500 MHz, DMSO): δ 155.20, 153.44, 144.23, 136.81, 133.17, 127.06, 125.97, 122.32, 118.56, 117.15, 116.40, 112.99, 110.78 ppm. HRMS calculated for C_15_H_11_NO_2_ 237.08 found 237.08.

*Synthesis of* 4-(naphthalen-2-yl)benzene-1,2-diol (**23**). To a mixture of 2-naphtylboronic acid (5.81 mmol), K_2_CO_3_ (5.81 mmol), and Pd(OAc)_2_ (0.048 mmol) in ethanol (15 ml) and water (5 ml) was slowly added 4-bromo-1,2-dimethoxybenzene (4.84 mmol). The mixture was stirred at room temperature for 18 h. To the resulting mixture was added water (80 ml), and then extracted with dichloromethane. The organic phase was separated, dried over Mg_2_SO_4_ and concentrated under reduced pressure to give a solid residue which was purified by flash chromatography (silica gel, hexane/ethyl acetate 5/1). The pure 2–(3,4-dimethoxyphenyl)naphthalene was obtained as white solid and used for the next reaction. Yield (%) = 93. ^1^H NMR (500 MHz, DMSO): δ 8.29 (d, *J* = 8.0 Hz, 1H), 8.12 (d, *J* = 8.0 Hz, 1H), 7.98 (d, *J* = 7.4 Hz, 1H), 7.82 (t, *J* = 8.0 Hz, 1H), 7.75 (t, *J* = 8.0 Hz, 1H), 7.61 (s, 1H), 7.45 (d, *J* = 8.2 Hz, 1H), 7.39 (d, *J* = 7.4 Hz, 1H), 7.12 (s, 1H), 7.02 (d, *J* = 8.2 Hz, 1H), 3.85 (s, 3H), 3.78 (s, 3H) ppm. ^13 ^C NMR (500 MHz, DMSO): δ 151.20, 148.19, 139.45, 135.45, 132.13, 127.00, 124.15, 119.32, 117.56, 116.03, 113.40, 112.26, 110.67, 108.65, 57.23 ppm. HRMS calculated for C_18_H_16_O_2_ 264.12 found 264.12.

To a stirred solution of 2–(3,4-dimethoxyphenyl)naphthalene (0.36 mmol) in dichloromethane (3 ml) at 0 °C was added boron tribromide 1 M solution in dichloromethane (6.00 mmol). After 1 h, the resulting mixture was stirred for 3 more hours at room temperature. The resulting mixture was the poured into ice water and treated with methanol (20 ml). The pale pink mixture was then extracted with dichloromethane and the organic phase, previously dried over anhydrous MgSO_4_, was evaporated to give compound **23** as a grey solid. Yield (%) = 89. ^1^H NMR (500 MHz, DMSO): δ 9.56 (bs, 1H), 9.38 (bs, 1H), 8.31 (d, *J* = 8.1 Hz, 1H), 8.15 (d, *J* = 8.1 Hz, 1H), 7.88 (d, *J* = 7.5 Hz, 1H), 7.83 (t, *J* = 8.1 Hz, 1H), 7.75 (t, *J* = 8.1 Hz, 1H), 7.62 (s, 1H), 7.47 (d, *J* = 8.0 Hz, 1H), 7.39 (d, *J* = 7.5 Hz, 1H), 7.22 (s, 1H), 7.00 (d, *J* = 8.0 Hz, 1H) ppm. ^13 ^C NMR (500 MHz, DMSO): 149.80, 148.00, 140.46, 136.41, 133.16, 128.00, 125.16, 118.82, 117.76, 116.27, 112.47, 112.16, 111.57, 108.65 ppm. HRMS calculated for C_18_H_12_O_2_ 236.08 found 236.08.

## Biology

### Expression and purification of recombinant HIV-1 RT

Wt and mutant heterodimeric RT were expressed essentially as previously described^29^^,^^30^.

### RNase H inhibitor analysis

IC_50_ values were determined as previously reported^31^ using an 18-nt 3′-fluorescein-labeled RNA annealed to a complementary 18-nt 5′-dabcyl-labeled DNA. To a 96-well plate was added 1 μL of each inhibitor (in DMSO), followed by 10 μL of the appropriate RT (15−80 ng/mL) in reaction buffer. Hydrolysis was initiated by adding 10 μL of RNA/DNA hybrid (2.5 μM). Final assay conditions were 50 mM Tris·HCl, pH 8.0, 60 mM KCl, 10 mM MgCl_2_, 1% DMSO, 150−800 ng RT, 250 nM substrate, and increasing concentrations of inhibitor. Wells containing only DMSO was used as negative control. Plates were incubated at 37 °C in a Spectramax Gemini EM fluorescence spectrometer for 10 min, and fluorescence (*l*_ex_= 485 nm; *l*_em_ = 520 nm) was measured at 1-min intervals such that linear initial rates could be measured in the presence (v_i_) and absence (v_o_) of inhibitor. Percent inhibition was calculated as 100 × (v_o_–v_i_)/v_o_ and plotted against log[I]. IC_50_ values were determined using Prism5 (GraphPad Software, San Diego, CA). All assays were performed in triplicate.

### Expression and purification of recombinant HIV-1 in and LEDGF

HIV IN was expressed as previously described^32–34^.

### HTRF in LEDGF-dependent assay

The IN LEDGF/p75-dependent assay measures inhibition of IN activity in the presence of LEDGF/p75 protein^35–37^. Briefly, 50 nM IN was pre-incubated with increasing concentration of compounds for 1 h at room temperature in reaction buffer containing 20 mM HEPES pH 7.5, 1 mM DTT, 1% Glycerol, 20 mM MgCl2, 0.05% Brij-35 and 0.1 mg/ml BSA. To this mixture, 9 nM DNA donor substrate (5′-ACAGGCCTAGCACGCGTCG-Biotin-3′ annealed with 5′-CGACGCGTGGTAGGCCTGT-Biotin3’) and 50 nM DNA acceptor substrate (5′-Cy5-ATGTGGAAAATCTCTAGCAGT-3′ annealed with 5′-Cy5- TGAGCTCGAGATTTTCCACAT-3′) and 50 nM LEDGF/p75 were added and incubated at 37 °C for 90 min. After incubation, 4 nM of Europium–Streptavidin was added to the reaction mixture and the HTRF signal was recorded using a Perkin Elmer Victor 3 plate reader using a 314 nm for excitation wavelength and 668 and 620 nm for the wavelength of the acceptor and the donor substrates emission, respectively.

### HIV-1 RNA-dependent DNA polymerase activity determination

HIV-1 RT-associated RDDP activity was measured as described^17^^,^^38^. Briefly, in 25 μL volume containing 60 mM Tris-HCl pH 8.1, 8 mM MgCl2, 60 mM KCl, 13 mM DTT, poly(A)-oligo(dT), 100 μM dTTP, and 6 ng wt RT. After enzyme addition, the reaction mixture was incubated for 30 min at 37 °C and stopped by addition of EDTA. Reaction products were detected by PicoGreen addition and measured with a Victor 3 (Perkin) at 502/523 nm. IC_50_ values were determined using GraphPad Prism version 6.01 software (GraphPad Software, Inc.; San Diego, CA). Figures were prepared with GraphPad Prism 6 version 6.01. All assays were performed in triplicate.

### Evaluation of MgCl_2_ coordination

The coordination properties for the compounds were determined as reported previously^39–41^. Briefly, compounds were solubilised in 1 ml of 96% ethanol at a final concentration of 100 μM. The UV–Vis spectrum was recorded from 200 to 600 nm before and after titration with increasing final concentrations of MgCl_2_, from 100 to 10 mM. UV–Vis spectra were recorded from 200 to 600 nm using an Ultrospec 2100 pro (Amersham Biosciences, Little Chalfont, UK) and spectra were plotted using SigmaPlot for Windows version 11.0 (Systat Software Inc., San Jose, CA). Colour legend indicates the final Mg^2+^ micromolar concentration within the sample.

### Differential scanning fluorimetry (ThermoFluor)

Thermal stability assays were performed according to Nettleship et al.^42^ To a LightCycler^®^480 96-well plate (Roche, Basel, Switzerland) was added 1 μL of 500 μM inhibitor in DMSO, followed by 49 μL of 300 nM HIV-1 RT in reaction buffer containing 20 mM HEPES, pH 7.5, 10 mM MgCl_2_, 100 mM NaCl, and a 1:1000 dilution of Sypro^®^ Orange dye (Invitrogen, Carlsbad, CA). The mixture was heated from 30 to 90 °C in increments of 0.2 °C. Fluorescence intensity was measured using excitation/emission wavelengths of 483 and 568 nm, respectively. Changes in protein thermal stability (ΔTm) upon inhibitor binding were analysed by using LightCycler^®^ 480 Software. All assays were performed in triplicate.

### Yonetani–Teorell analysis

Yonetani–Theorell analysis^43^ was performed as reported^44^. Briefly, HIV RT-associated RNase H activity was measured in 100 µL reaction volume containing 50 mM Tris–HCl buffer pH 7.8, 6 mM MgCl_2_, 1 mM dithiothreitol (DTT), 80 mM KCl, 0.25 µM hybrid RNA/DNA 5′-GAUCUGAGCCUGGGAGCU-Fluorescin-3′ (HPLC, dry, QC: Mass Check) (available from Metabion) 5′-Dabcyl-AGCTCCCAGGCTCAGATC-3′(HPLC, dry, QC: Mass Check), and different amount of enzymes according to a linear range of dose-response curve: 20 ng of WT RT, and increasing concentrations of inhibitor (diluted) in water and incubated for 1 h at 37 °C, at 200 rpm. Products were measured with a multilabel counter plate reader Victor 3 (Perkin Elmer model 1420-051) equipped with filters for 490/528 nm (excitation/emission wavelength). A calibration curve for the fluorescein signal was generated in parallel, calculating pmols of product generated by the reaction. The reciprocal of the reaction product was plotted *vs.* the compound concentration a different condition using the Sigma-Plot software.

### Antiviral activity

Antiviral activity of selected thienopyrimidinones was determined *via* the 2,3-bis[2-methoxy-4-nitro-5-sulfophenyl]-5-[(phenylamino)carbonyl]-2H tetrazolium hydroxide (XTT)-based cell viability assay of Weislow et al.^45^, using the HIV-1RF isolate and human T-cell line CEM-SS.

## Results and discussion

### Chemistry

**Scheme 1.**
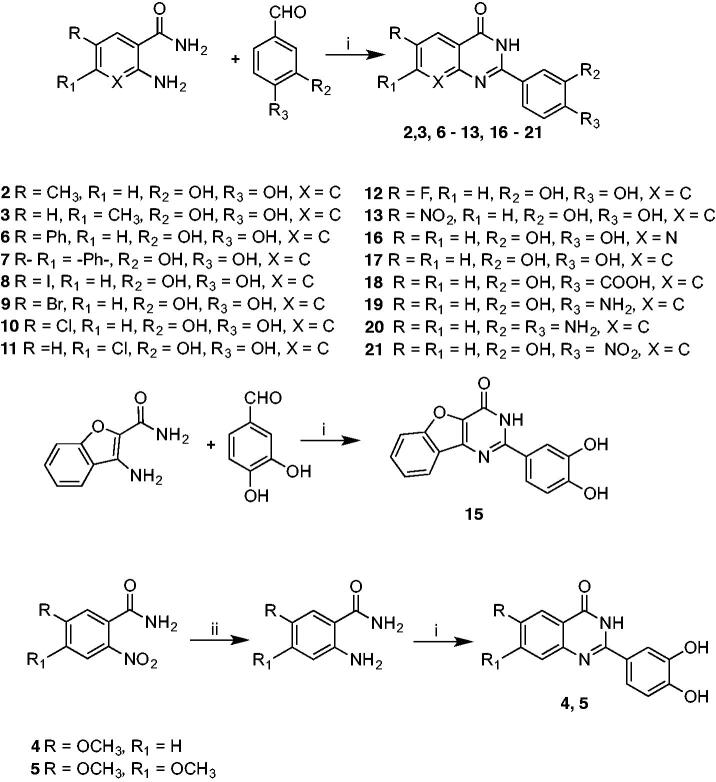
Synthetic protocol for compounds **2**–**13** and **15**–**21**. Reagents and conditions: i) I_2_/CH_3_CN, rt, 6 h. ii) SnCl_2_/HCl 37%,−5 °C (1 h), rt (30 h).

As reported in Scheme 1, compounds **2**, **3, 6 − 13**, **15 − 21** were synthesised by a one-pot direct oxidative condensation of the appropriate commercially available 2-amino arylamide with the 3,4-disubstituted benzaldehyde in the presence of molecular iodine^46^. After 6 h, the reaction, carried out in acetonitrile at room temperature, afforded the final aryl pyrimidinone derivative in good yields. Synthesis of quinazolinones **4** and **5** required synthesising the 2-amino-5-methoxybenzamide and 2-amino-4,5-dimethoxybenzamide. Starting from the corresponding 2-nitrobenzamide, we carried out a reduction of the nitro group^47^ in concentrated HCl in the presence of SnCl_2_, obtaining the corresponding mono or dimethoxy −2-amino arylamide as pure product.

**Scheme 2.**
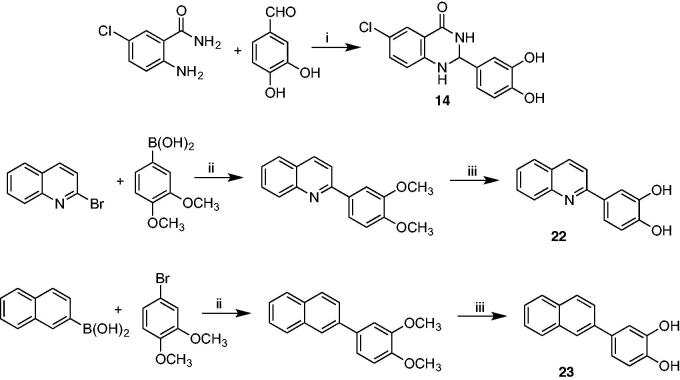
Synthetic protocol for compounds **14**, **22,** and **23**. Reagents and conditions: i) CH_2_Cl_2_/CH_3_CN, reflux, 40 h. ii) Pd(OAc)_2_/K_2_CO_3_, EtOH/H_2_O 3/1, rt (18 h). iii) BBr_3_ 1 M in CH_2_Cl_2_, CH_2_Cl_2_, 0 °C (1 h), rt (3 h).

In contrast, cyclisation^48^ (Scheme 2) of 2-amino-6-chlorobenzamide with 3,4-dihydroxybenzaldehyde carried out at reflux in a mixture of dichloromethane/acetonitrile gave the racemic 6-chloro-2–(3,4-dimethoxyphenyl)-2,3-dihydroquinazolin-4(1*H*)-one **14**, which is more flexible than the corresponding derivative **10**. Moreover, it adds a stereocenter on C-2, making the molecule chiral. Synthesis of compounds **22** and **23** started with a Suzuki C-C coupling reaction between the appropriate arylboronic acid and the proper aryl bromide under basic conditions and in the presence of Pd(OAc)_2_ as catalyst^49^. Subsequent demethylation was performed using BBr_3_ as demethylating agent in dichloromethane^50^.

### Biology

All compounds were tested for inhibitory activity against both wt and p66/p51 C280A RT. In fact, p51 residue Cys280 is crucial for binding of thienopirimidinones^51^. Table 1 provides IC_50_ values for the first panel of quinazolinone derivatives. It is immediately evident that every substitution gave rise to compounds with submicromolar activity, equally effective on both RT wild type and p66/p51 C280A mutant.

Interestingly, steric hindrance of the chosen substituents seemed not to affect RNase H activity. In fact, compounds **6** (IC_50_ = 0.37 μM) and **2** (IC_50_ = 0.41 μM) share the same potency. Conversely, compound **7** (IC_50_ = 1.10 μM) demonstrated that adding rigidity to the molecule by introducing another aromatic ring ortho condensed to the quinazolinone core impaired the activity while replacing naphthalene with benzofuran as in **15** (IC50 = 0.64 μM) was only slightly detrimental to inhibitory potency. Noteworthy, electron withdrawing groups, and in particular halogens in position 6, provided the most active compounds, with **8** being the best compound (IC_50_ = 0.15 μM) of the entire series of derivatives. Finally, the 2–(3,4-dihydroxyphenyl) unit was demonstrated to be crucial for the inhibitory activity similarly to thienopyrimidinones. In fact, as reported in Table 2, replacing one or both OH groups with –COOH, NH_2_, or NO_2_ led to a complete loss of activity.

**Table 2.** Inhibition of HIV-1 RNase H activity by compounds **18**–**21**. 
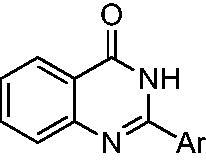

Compound	Ar	IC_50_ (µM)
		WT
**18**		13.6 ± 0.3
**19**		>50
**20**		>50
**21**		>50

We also explored the role of the pyrimidinone moiety. First, in order to determine the importance of the rigidity of the quinazolinone core, we decided to synthesise compound **14**, in which removal of the double bond between N-1 and C-2 gave more flexibility to the pyrimidinone ring and introduced a stereocenter on C-2. Since the synthetic pathway was not stereoselective, we obtained and subsequently tested, compound **14** as a racemic mixture. As shown in Table 1, this structural modification did not affect RNase H inhibitory activity on both wt and p66/p51 C280A mutant RTs. Encouragingly, this modification rendered quinazoline **14** more selective for RNase H activity than **9** (Table 4) and prompted us to explore a stereoselective synthetic approach that will be the subject of a future paper.

As shown in Table 3, removing the amide unit gave rise to 2–(3,4-dihydroxyphenyl) quinoline **22** with almost the same inhibitory potency of 2–(3,4-dihydroxyphenyl)- quinazolin-4(3*H*)-one **17** against wt RT (IC_50_ = 2.9 uM)^26^. Noteworthy, **22** inhibited p66/p51 C280A mutant RT RNase H activity 4-fold more potently than wt RT (IC_50_ = 0.61 μM). Substituting the pyridine core with its simplest bioisoster, a benzene ring, generated compound **23**, 5-fold more potent than the reported compound **17**. However, **23** inhibited p66/p51 C280A mutant RT RNase H activity with the same potency of the wt enzyme (Table 3).

**Table 3.** Inhibition of wild type and p66/p51C280A mutant HIV-1 RNase H activity by compounds **22** and **23**.

Compound		IC_50_ (µM)	
		WT	C280A
**22**		2.9 ± 0.89	0.61 ± 0.22
**23**		0.71 ± 0.03	1.2 ± 0.04

### Inhibitory activity against RNA-dependent DNA polymerase (RDDP) and integrase (IN)

In order to determine the specificity of our quinazolinone-based derivatives, we tested their effect on RDDP and IN activities. With respect to RDDP function, all compounds were assayed using efavirenz, a non-nucleoside RT inhibitor, as positive control. Data of Table 4 indicate that all the quinazolinone derivates, albeit with a different degree, were poorly active against RDDP with IC_50_ values ranging from 23 to more than 100 µM. Thus, they are highly selective RNase H inhibitors.

**Table 4.** Effect of compounds **2**–**17** on HIV-1 RDDP and IN functions.

Compound	IC_50_ (µM)	
	RDDP	IN
**2**	>100	4.3 ± 1.5
**3**	60 ± 9	2.35 ± 0.44
**4**	57 ± 3	8.5 ± 0.3
**5**	>100	6.5 ± 2.5
**6**	63 ± 11	89 ± 10
**7**	23.4 ± 0.5	31.5 ± 6.5
**8**	26.7 ± 2.2	3.4 ± 0.83
**9**	46 ± 3	7 ± 2
**10**	30.5 ± 0.4	3.4 ± 0.6
**11**	>100	7.2 ± 0.7
**12**	>100	6.61 ± 0.93
**13**	99 ± 1	23.18 ± 0.8
**14**	>100	>100
**15**	25.3 ± 6.3	5.35 ± 0.35
**16**	38 ± 4	0.69 ± 0.19
**17**	70 ± 1	1.44 ± 0.40
**EFV**	0.010 ± 0.005	ND
**RAL**	ND	0.055 ± 0.002

Since the catechol moiety has been identified in a variety of HIV-1 IN inhibitors^52^^,^^53^, we also tested our compounds against this target, using the strand-transfer inhibitor raltegravir, as positive control. Almost all quinazolinones were moderately active on HIV-1 IN, displaying, and IC_50_ range of 2.3–89 µM, showing high selectivity for RNase H. Remarkably, the pyridine core was subtly deleterious for RT activity but powerful in IN function suppression. In fact, compounds **16** and **17** showed a different trend. In particular, these compounds inhibited IN activities in the presence of LEDGF/p75 protein with IC_50_ values of 0.69 and 1.44 µΜ, respectively. Moreover, these derivatives exhibited a higher selectivity against IN.

### Investigation of mode of action

Typically, RNase H active site inhibitors chelate the catalytically crucial Mg^2+^ ions. Since catechol-containing compounds usually display metal-chelating properties; we performed UV spectrometric analysis to determine the possible involvement of Mg^2+^ cofactors in the inhibition mode of our quinazolinones. In this respect, a magnesium effect was examined at increasing concentrations of the divalent metal (Supplementary Material). Results showed that the presence of the Mg^2+^ failed to shift the peak of absorbance of the compounds spectra, excluding the involvement of Mg^2+^ cofactor coordination on the mode-of-action of compounds. Only compound **2** showed a hyperchromic effect at the highest concentration tested.

**Figure 2.**
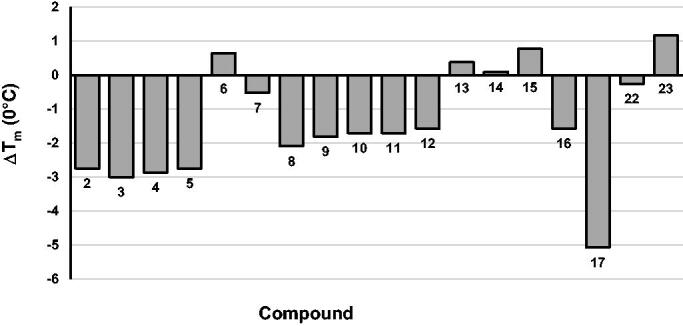
Effect of compounds **2**–**17**, **22**, and **23** on the thermal stability of p66/p51 HIV-1 RT. Tm values are the average of triplicate analysis.

To gain additional evidence, thermal denaturation experiments were carried out. Differential scanning fluorimetry was the technique of choice, as can determine the effect of an inhibitor on RT stability by measuring changes in its melting temperature (T_m_)^54^. Results graphically reported in Figure 2, indicate that almost all tested inhibitors destabilise the RT heterodimer stability, i.e. they decrease the T_m_. Since it had been demonstrated that compounds that bind in the proximity of the p66/p51 interface usually reduce RT T_m_^11^, we can assume that present quinazolinones, similarly to thienopyrimidinones, might act as allosteric inhibitors.

**Figure 3.**
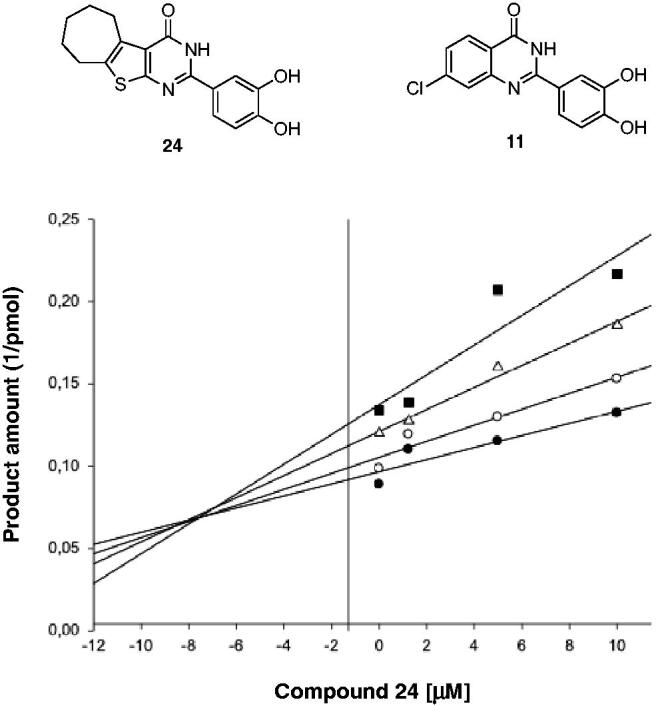
Yonetani–Theorell analysis. Combination of compound **11** and **24** on HIV-1 RNase H activity. HIV-RT was incubated in the presence of **24** alone (●) or combined with increasing concentrations of compound **11**: 2,5 µM (O); 5 µM(Δ) and 10 µM (■).

To confirm this hypothesis, we performed a Yonetani-Theorell analysis of simultaneous action of compound **11** *vs.* the 2–(3,4-dihydroxyphenyl)-3,5,6,7,8,9-hexahydro-4*H*-cyclohepta[4,5]thieno[2,3-*d*]pyrimidin-4-one **24**^12^ on HIV-1 RT-associated RNase H activity. Such an analysis reveals whether simultaneous binding (or inhibition) of two compounds is possible or not. Results showed that compound **11** and compound **24** inhibition are not kinetically mutually exclusive (Figure 3), suggesting that they may bind simultaneously to RT. However, it is worth noting that the calculated interaction constant α had a value of greater than 1, suggesting a negative interference between the two compounds (i.e. compound binding **11** has a negative influence on compound **24** binding, and vice versa).

### Antiviral activity

The ability of all compounds to inhibit HIV-1 virus replication was evaluated. Only compounds **3**, **8**, **10**, **11,** and **23**, showed a modest antiviral activity within low micromolar range. Unfortunately, they displayed very low SI values, demonstrating to be highly cytotoxic. In fact, the most active compound in cell-based assays, compound **8**, showed a very small selectivity index (SI) (EC_50_ = 8.3 µM, CC_50_ = 17 µM, SI = 2). Similar results were obtained for **3** (EC_50_ = 12 µM, CC_50_ = 25 µM, SI = 2) and **23** (EC_50_ = 14 µM, CC_50_ = 26 µM, SI = 2). Interestingly, the simple substitution of an iodine atom with a chlorine in **10** displayed a slightly better SI (EC_50_ = 5.4 µM, CC_50_ = 22 µM, SI = 4). Ring walking of the chlorine from position 6–7 as in **11**, reduced the SI (EC_50_ = 1.8 µM, CC_50_ = 6 µM, SI = 3). All other compounds were inactive (data not shown).

## Conclusions

The isosteric replacement approach allowed us to synthesise a series of 22 quinazolinone-based derivatives. All compounds were equally effective on both wt RT and p66/p51 C280A mutant, unable to chelate the Mg^2+^ ions, and in most cases destabilised the RT heterodimer by decrease its T_m_. These combined findings prompted us to hypothesise that they are not active site, but most likely allosteric, and in particular RT heterodimer interface inhibitors. Yonetani–Theorell analysis showed that compound **11** and the reference thienopyrimidinone **24** are not kinetically mutually exclusive and the value of the calculated interaction constant *α* indicated a negative interference between the two compounds. Thus, they may bind simultaneously to different, but probably close, RT allosteric sites since compound **11** binding has a negative influence on compound **24** binding, and vice versa.

Moreover, all compounds were highly selective for RNase H function, since they were poorly active on RDDP activity and moderately active on HIV-1 IN. The exception to this was compounds **16** and **17**, which exhibited a higher selectivity against IN.

Interestingly, compounds **3**, **8**, **10**, **11,** and **23** demonstrated to suppress HIV-1 replication in a cell-based assay albeit with a modest antiviral activity, within the low micromolar range, but unfortunately showed to be cytotoxic, too.

As previously reported for other allosteric RNase H inhibitors^11^^,^^55^, the presence of the catechol moiety was essential for the inhibitory activity of our compounds. However, it cannot be discarded that, because of its redox reactivity, it might be in part responsible for the poor antiviral activity and cytotoxicity, too. In fact, toxicity of catechol-containing drugs is often related to the ability of semiquinone radical, the catechol oxidised form, to participate in production of a superoxide anion radical deleterious for proteins and cell membranes^56^^,^^57^.

In summary, bioisosteric replacement of the thiophene with a benzene ring proved a worthwhile strategy since all compounds reported here retained their inhibitory activity against both RT wild type and p66/p51 C280A mutant. Thus, the quinazolinone-based derivatives reported herein may be promising hit molecules for the development of a novel generation of selective RNase H allosteric inhibitors without the liability of the thiophene series.

## Supplementary Material

Supplemental Material
