# Supplementary material for: Scaffold hopping and optimisation of 3’,4’-dihydroxyphenyl- containing thienopyrimidinones: synthesis of quinazolinone derivatives as novel allosteric inhibitors of HIV-1 reverse transcriptase-associated ribonuclease H
Source: J Enzyme Inhib Med Chem. 2020 Nov 3;35(1):1953–63. doi: 10.1080/14756366.2020.1835884 (PMC7646544; doi:10.1080/14756366.2020.1835884)
Supplement: Supplemental Material [file IENZ_A_1835884_SM8795.pdf]

Scaffold Hopping and Optimization of 3’,4’-Dihydroxyphenyl- containing Thienopyrimidinones: Synthesis of Quinazolinone Derivatives as Novel Allosteric Inhibitors of HIV-1 Reverse Transcriptase-Associated Ribonuclease H

Graziella Tocco[a]*, Francesca Esposito[a], Pierluigi Caboni[a], Antonio Laus[a], John A. Beutler[b], Jennifer A. Wilson[b], Angela Corona[a], Stuart F.J. Le Grice[c] and Enzo Tramontano[a]

[a]Department of Life and Environmental Sciences, University of Cagliari, Cittadella Universitaria di Monserrato, Monserrato, Cagliari 09042, Italy. [b]Molecular Targets Program, National Cancer Institute, Frederick, Maryland 21702, USA. [c]Basic Research Laboratory, National Cancer Institute, Frederick, Maryland 21702, USA.

*corresponding author

Graziella Tocco, Department of Life and Environmental Sciences, Unit of Drug Science, University of Cagliari, Cittadella Universitaria di Monserrato, Monserrato, Cagliari 09042, Italy. toccog@unica.it

Supplementary material:

Evaluation of $MgCl_2$ Coordination

The coordination properties for the compounds was determined as reported previously. Briefly, compounds were solubilized in 1mL of 96% ethanol at a final concentration of 100 μM. The UV-Vis spectrum was recorded from 200 nm to 600 nm before and after titration with increasing final concentrations of $MgCl_2$, from 100 μM to 10 mM. UV-Vis spectra was recorded from 200 to 600 nm using a Ultrospec 2100 *pro* (Amersham Biosciences) and spectra were plotted using SigmaPlot for Windows version 11.0. Color legend indicates the final Mg^{2+} micromolar concentration within the sample.

Compound **2** coordination Mg^{2+}

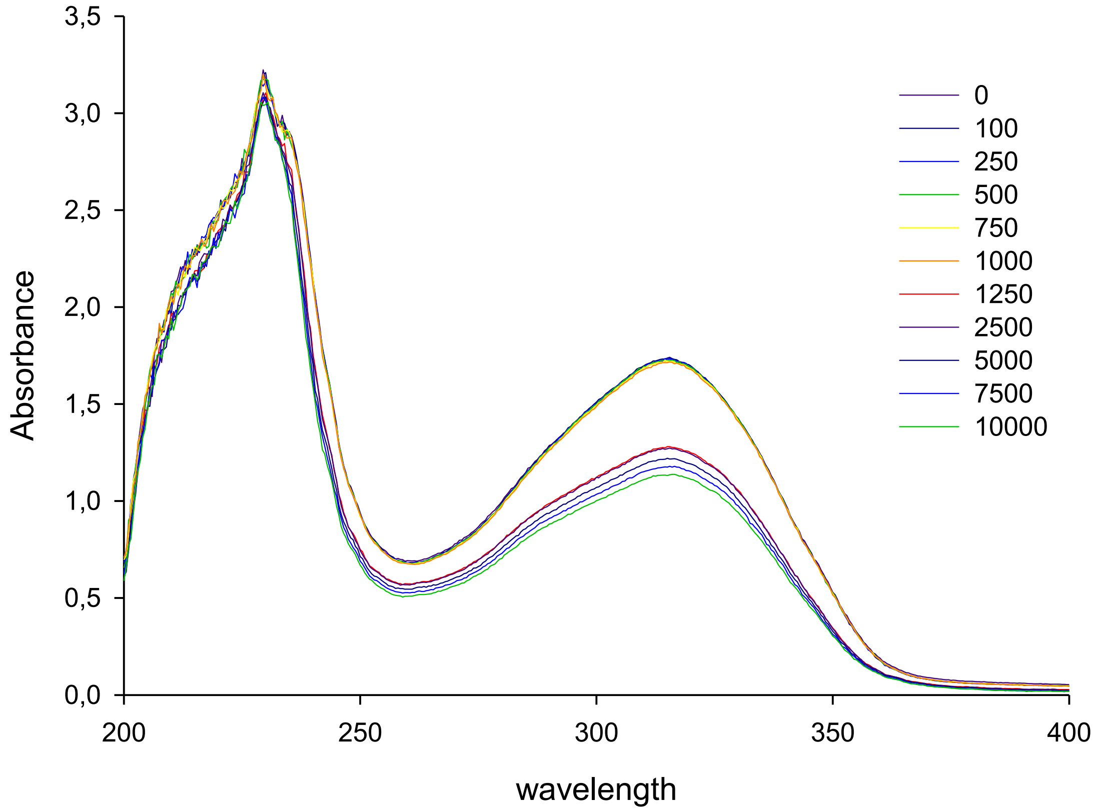

Compound **3** coordination Mg^{2+}

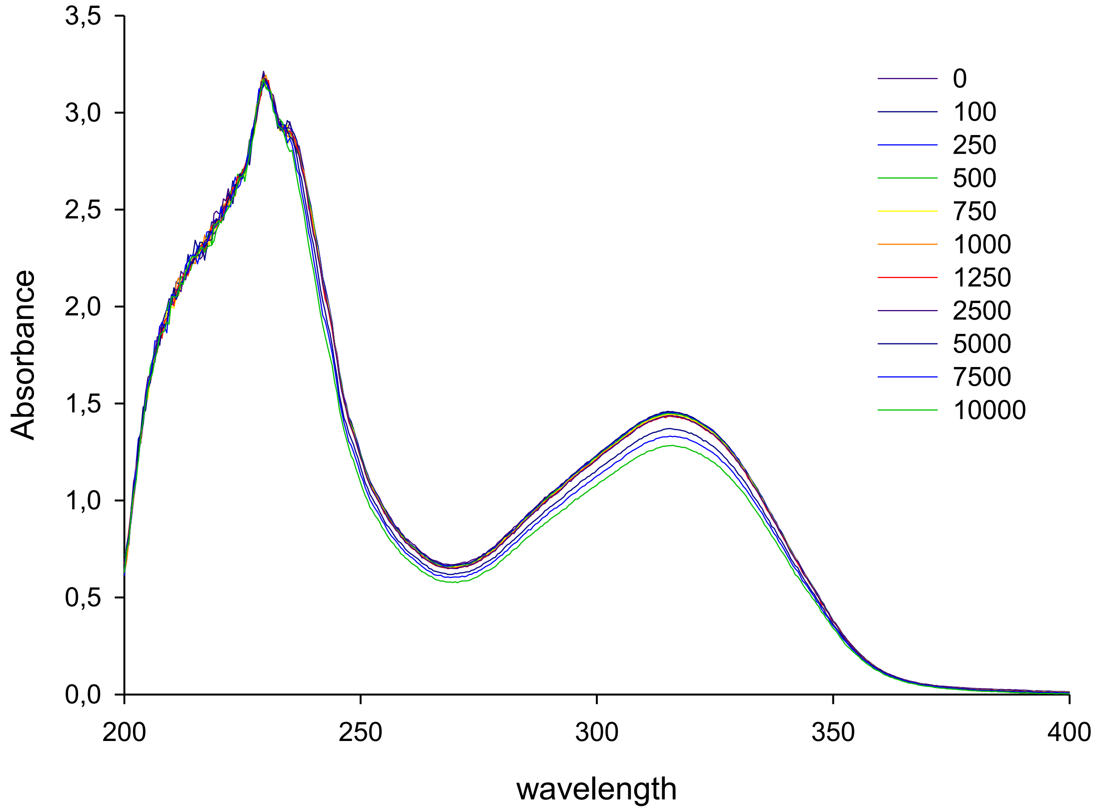

Compound **4** coordination Mg^{2+}

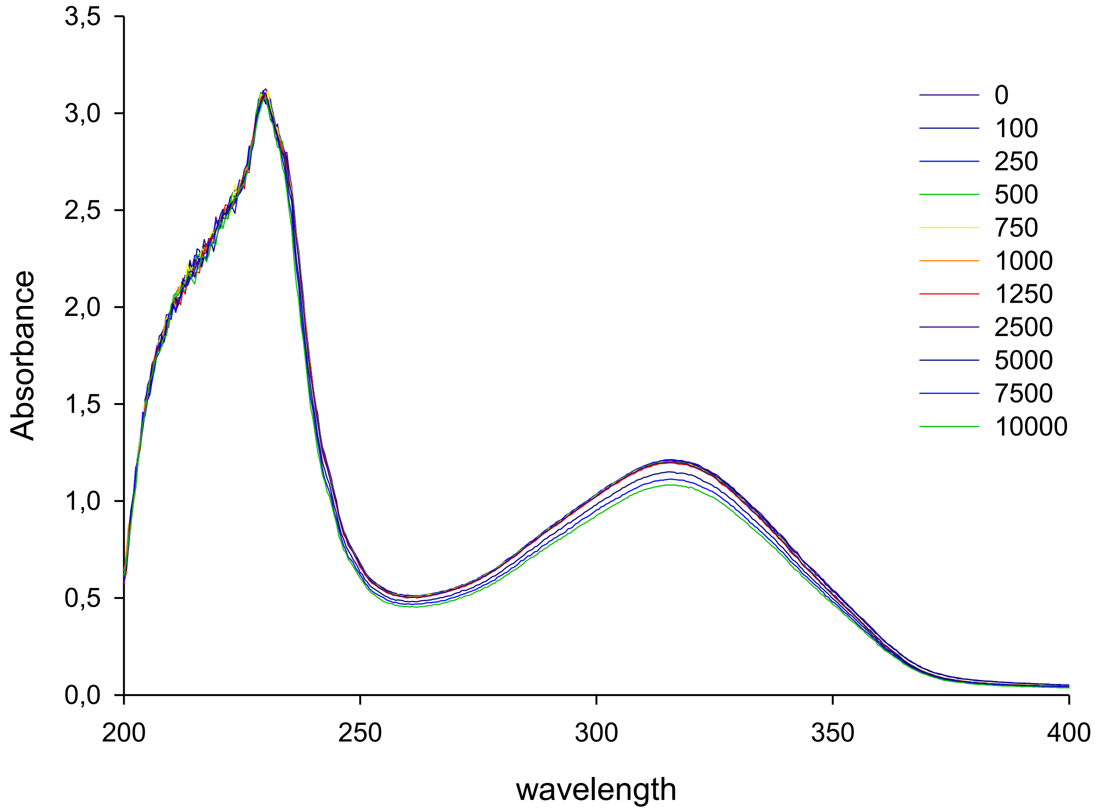

Compound **5** coordination Mg^{2+}

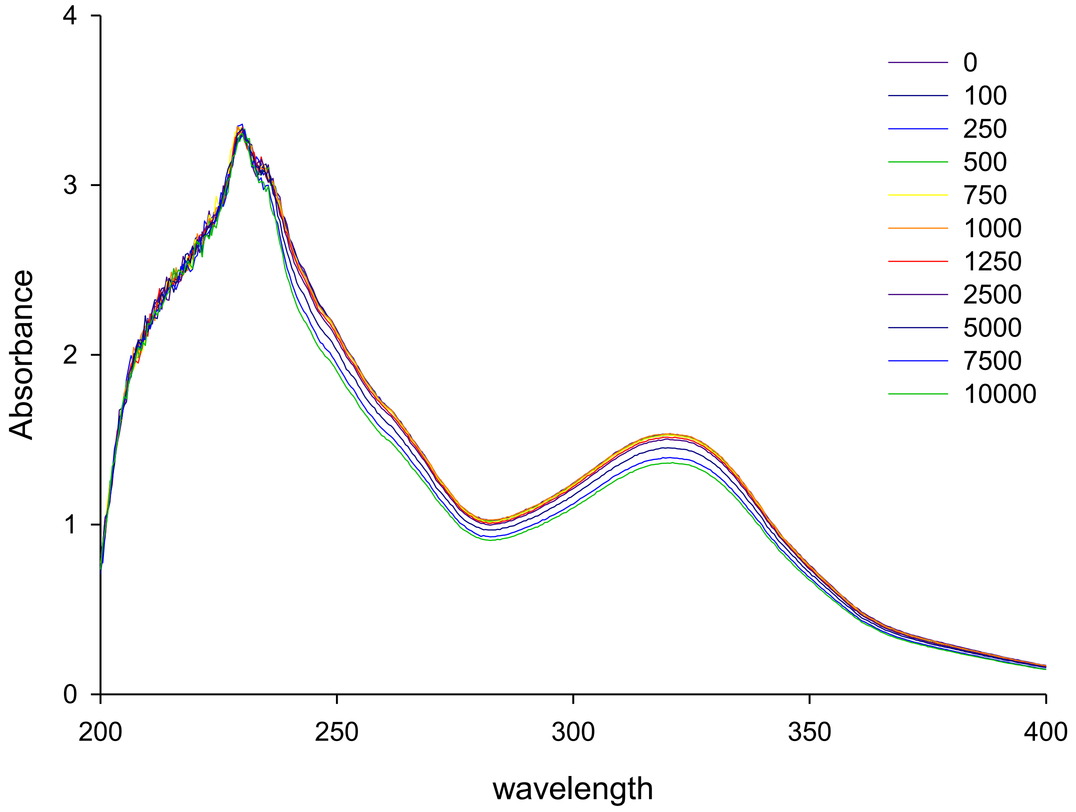

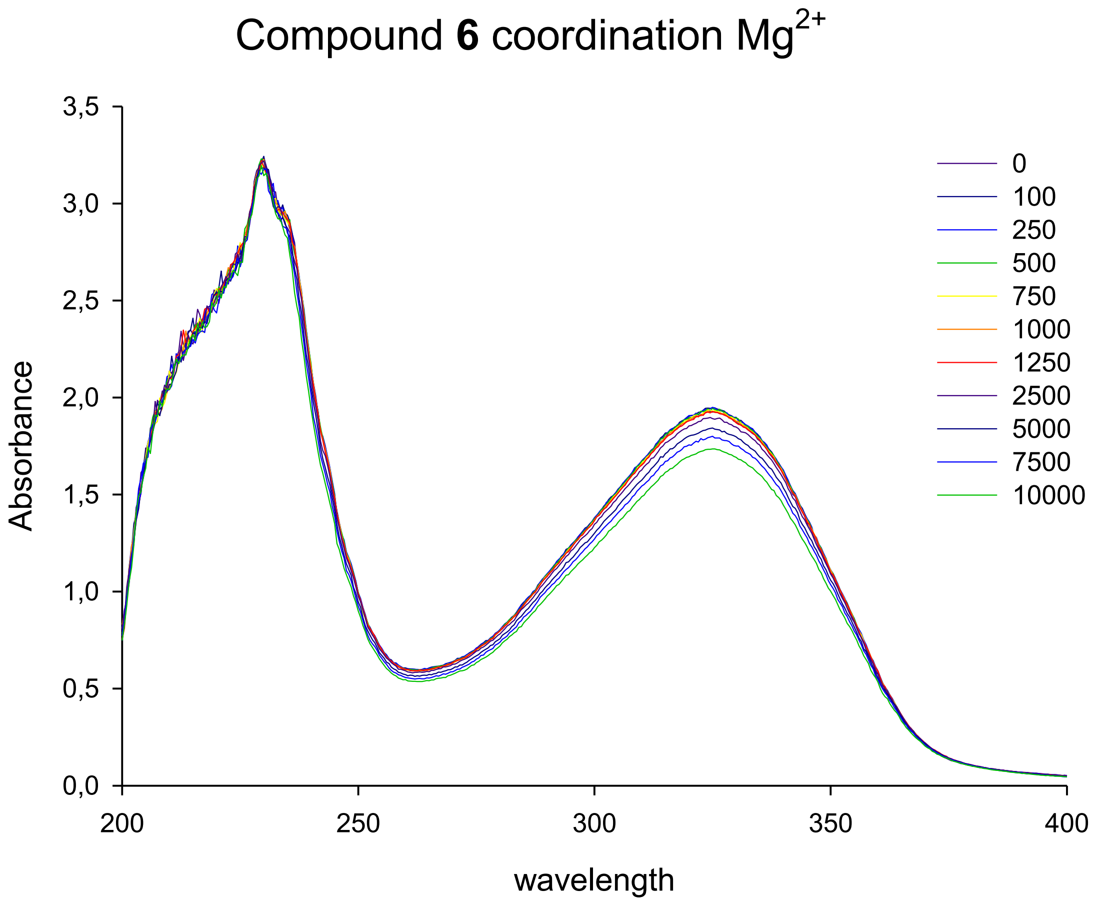
Compound **6** coordination Mg^{2+}
Absorbance
wavelength
0
100
250
500
750
1000
1250
2500
5000
7500
10000
3,5
3,0
2,5
2,0
1,5
1,0
0,5
0,0
200
250
300
350
400

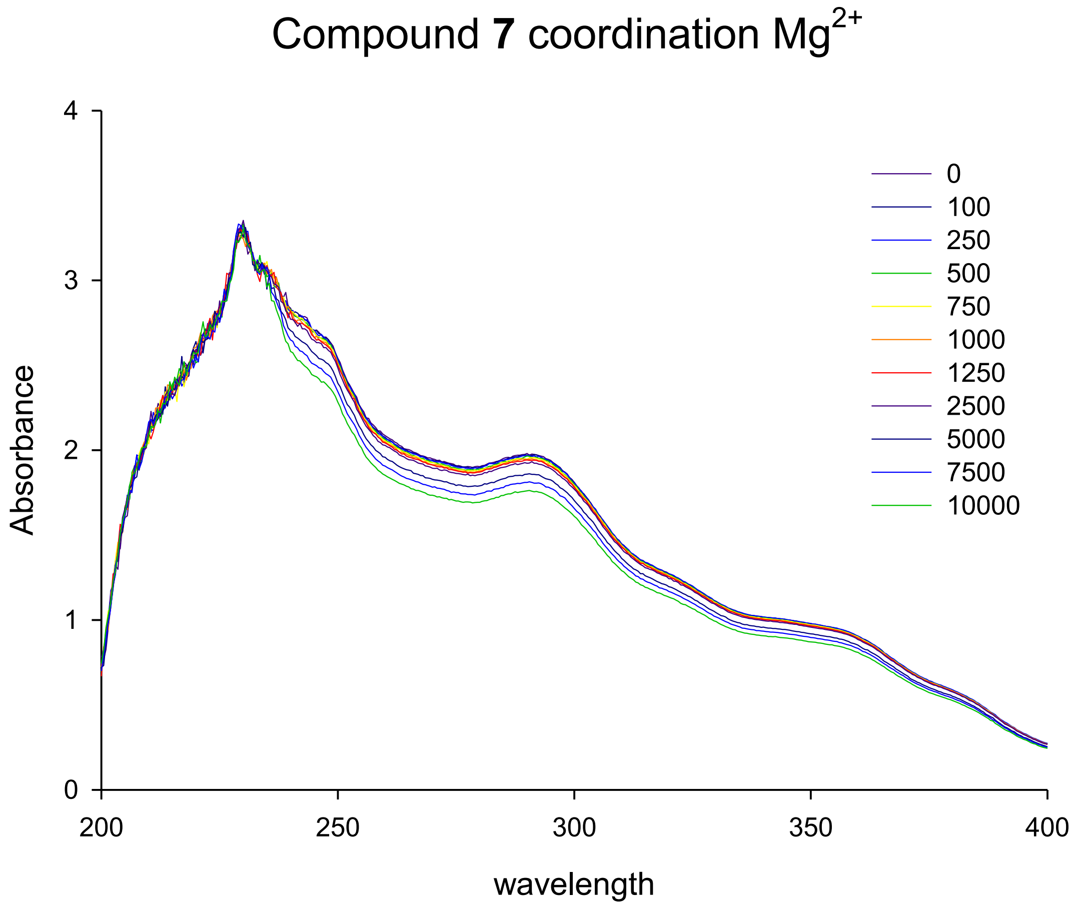
Compound **7** coordination Mg^{2+}
Absorbance
wavelength
0
100
250
500
750
1000
1250
2500
5000
7500
10000
4
3
2
1
0
200
250
300
350
400

Compound **8** coordination Mg^{2+}

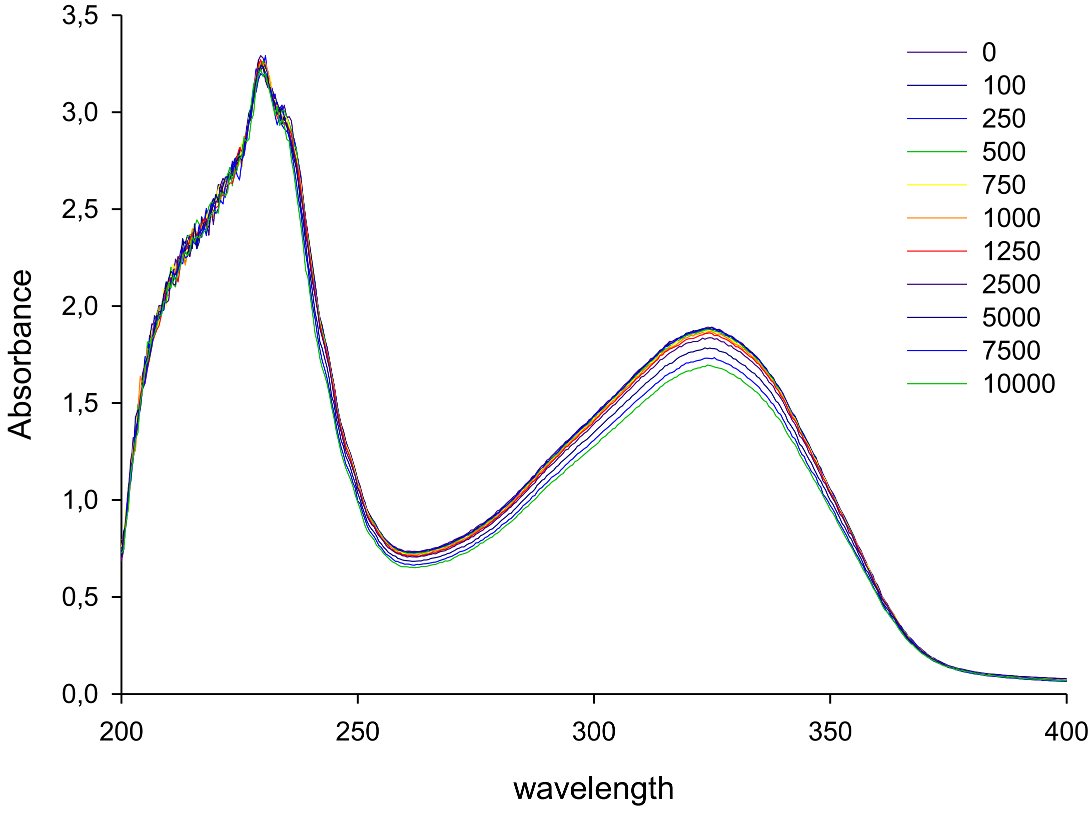

Compound **9** coordination Mg^{2+}

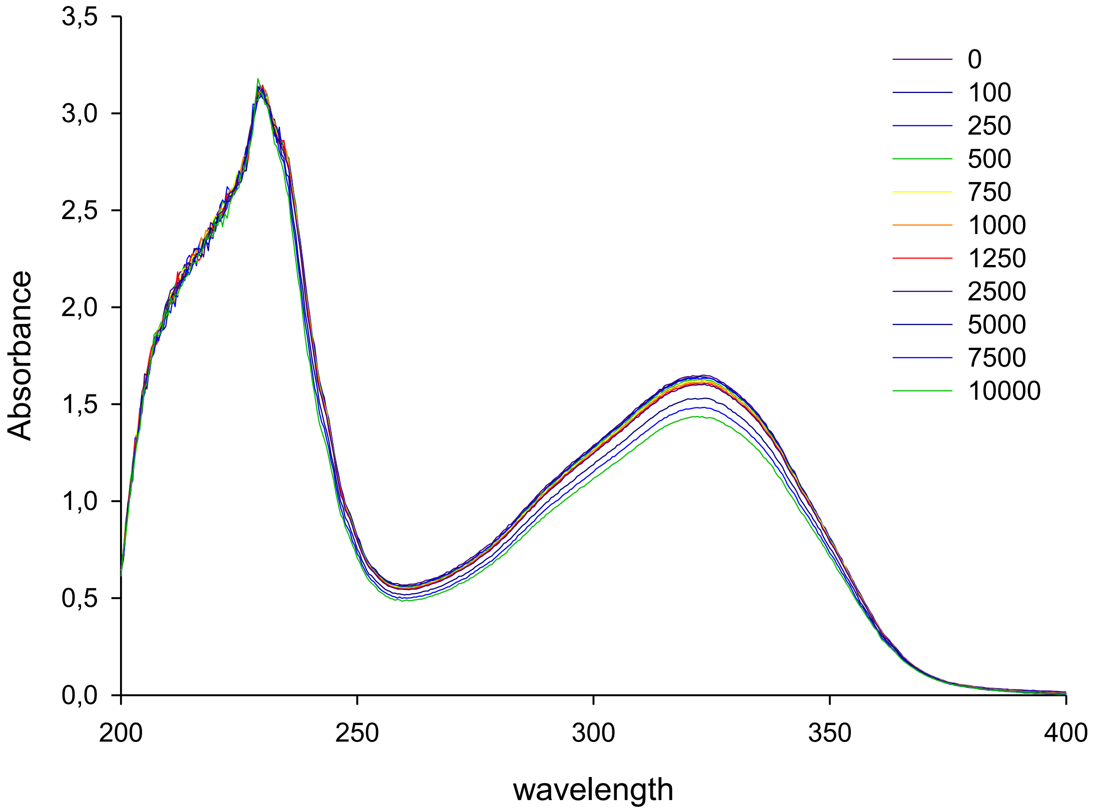

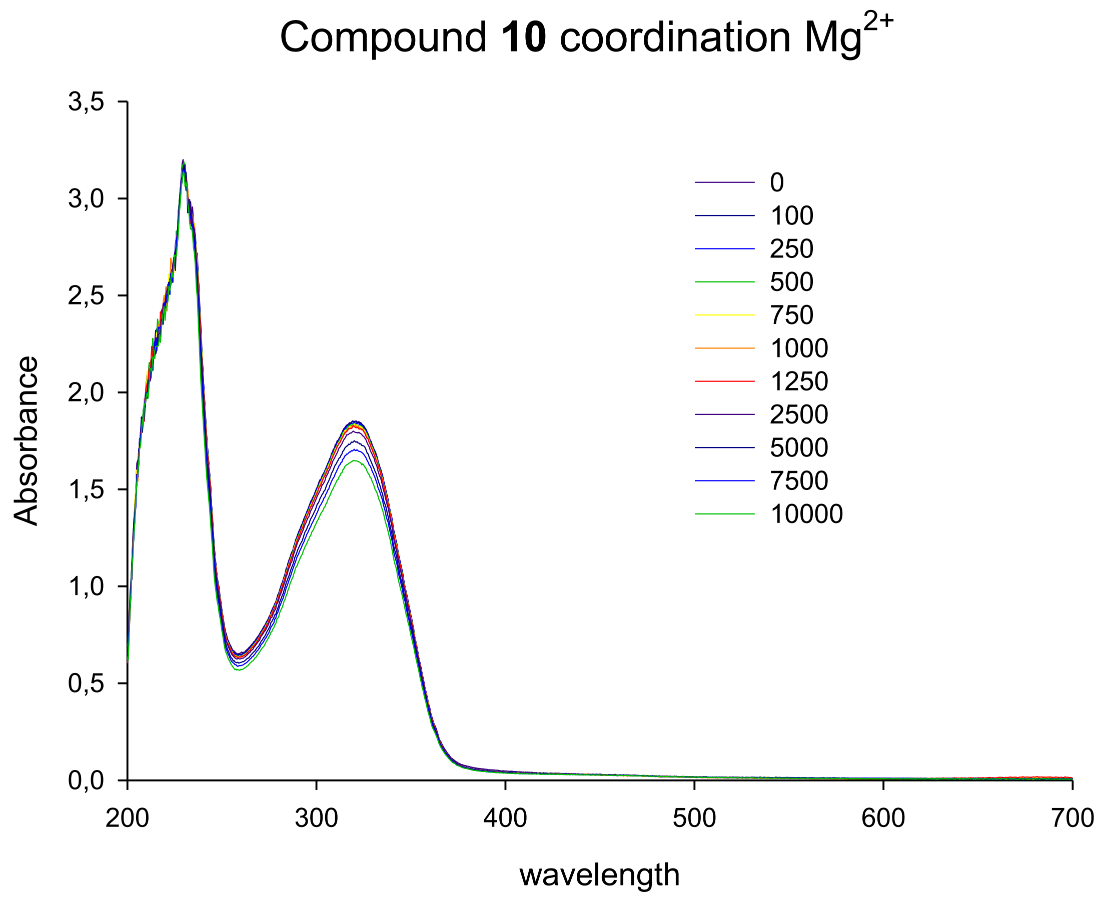

Compound **10** coordination Mg^{2+}
Absorbance
3,5
3,0
2,5
2,0
1,5
1,0
0,5
0,0
200
300
400
500
600
700
wavelength
0
100
250
500
750
1000
1250
2500
5000
7500
10000

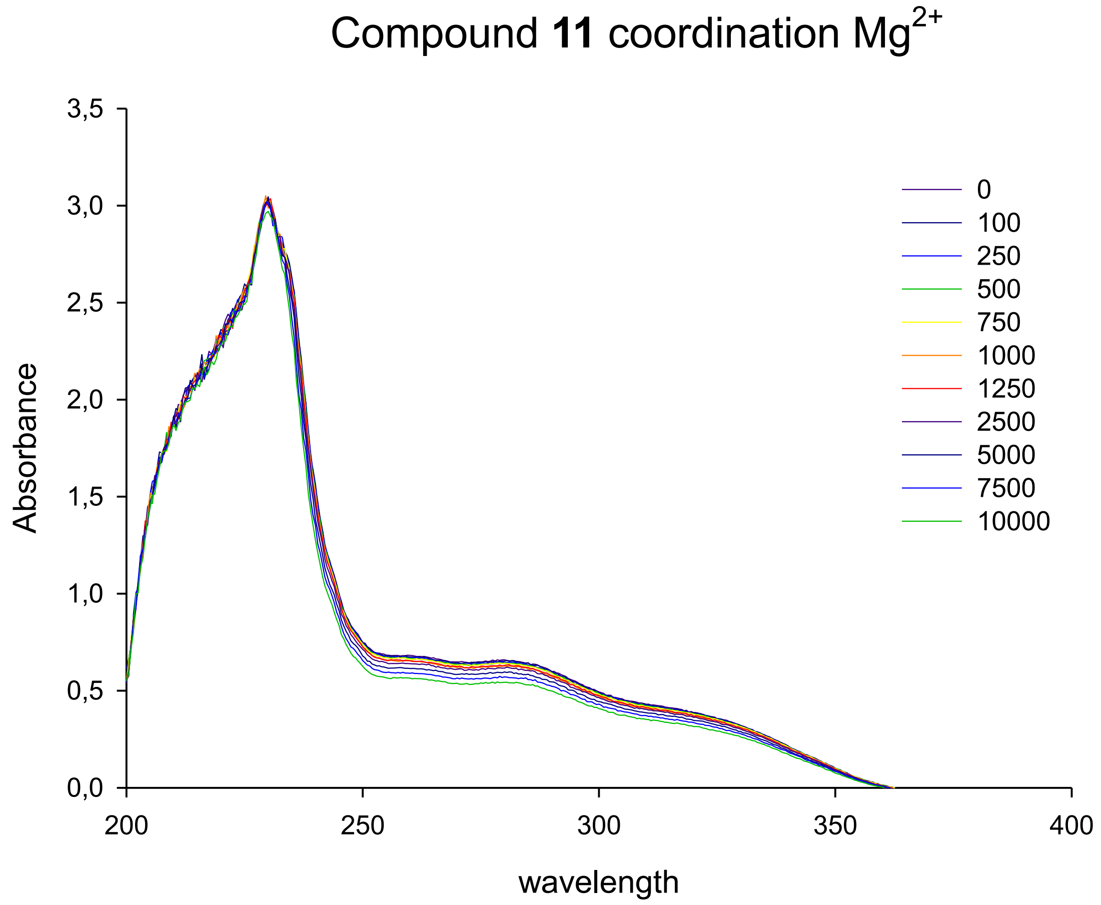

Compound **11** coordination Mg^{2+}
Absorbance
3,5
3,0
2,5
2,0
1,5
1,0
0,5
0,0
200
250
300
350
400
wavelength
0
100
250
500
750
1000
1250
2500
5000
7500
10000

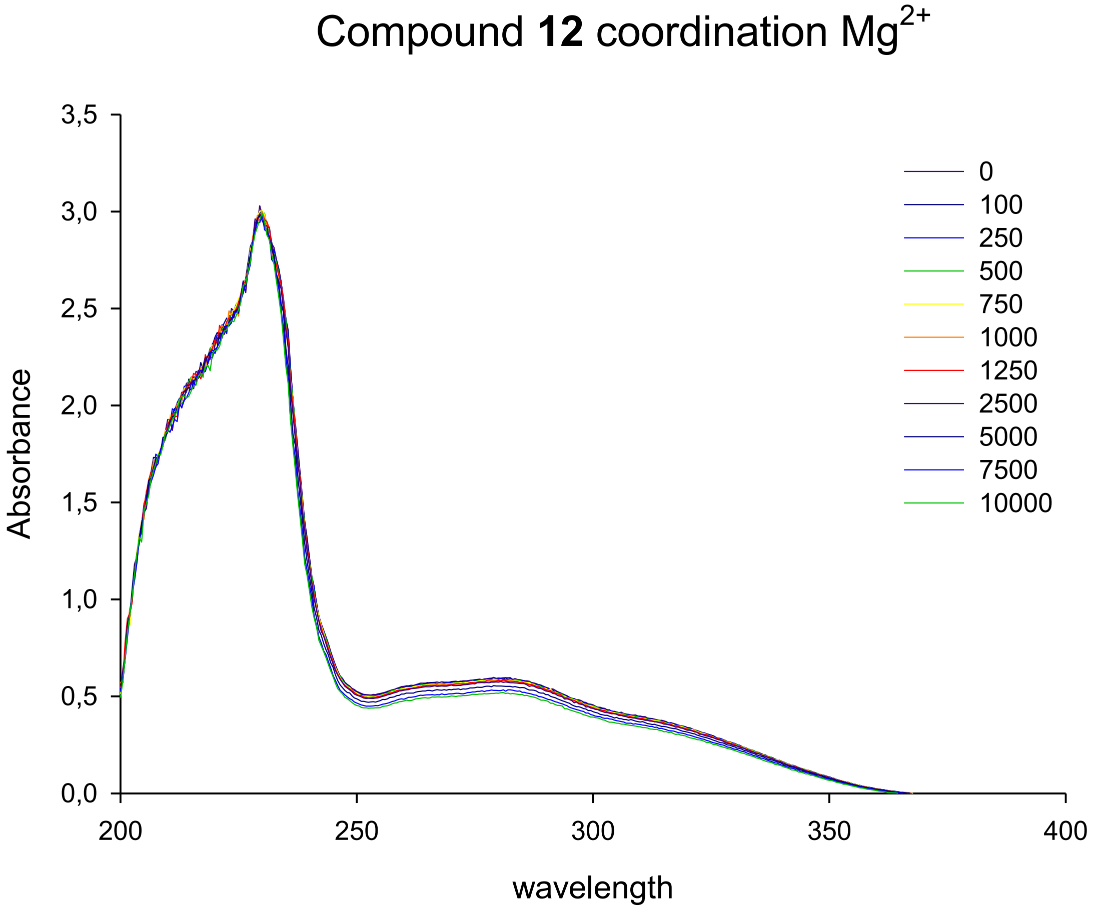
Compound 12 coordination Mg2+
Absorbance
wavelength
3,5
3,0
2,5
2,0
1,5
1,0
0,5
0,0
200
250
300
350
400
0
100
250
500
750
1000
1250
2500
5000
7500
10000

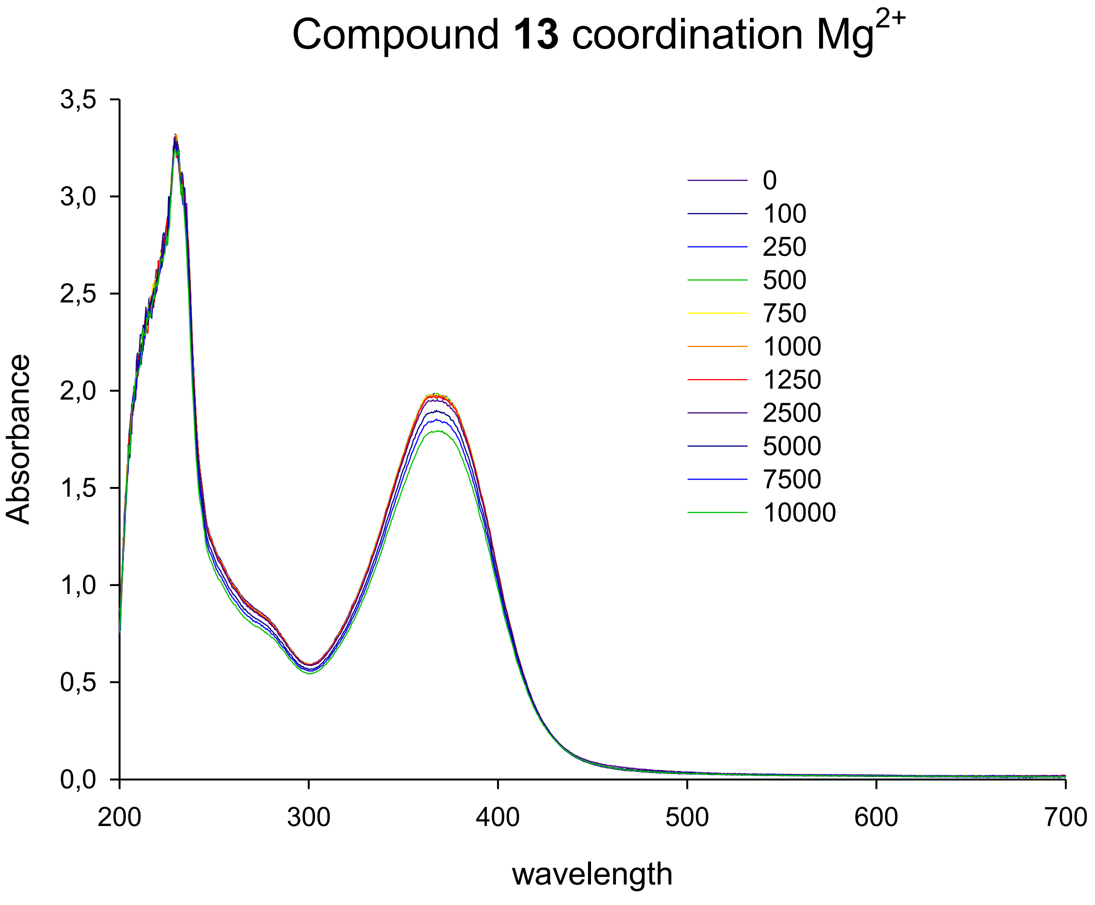
Compound 13 coordination Mg2+
Absorbance
wavelength
3,5
3,0
2,5
2,0
1,5
1,0
0,5
0,0
200
300
400
500
600
700
0
100
250
500
750
1000
1250
2500
5000
7500
10000

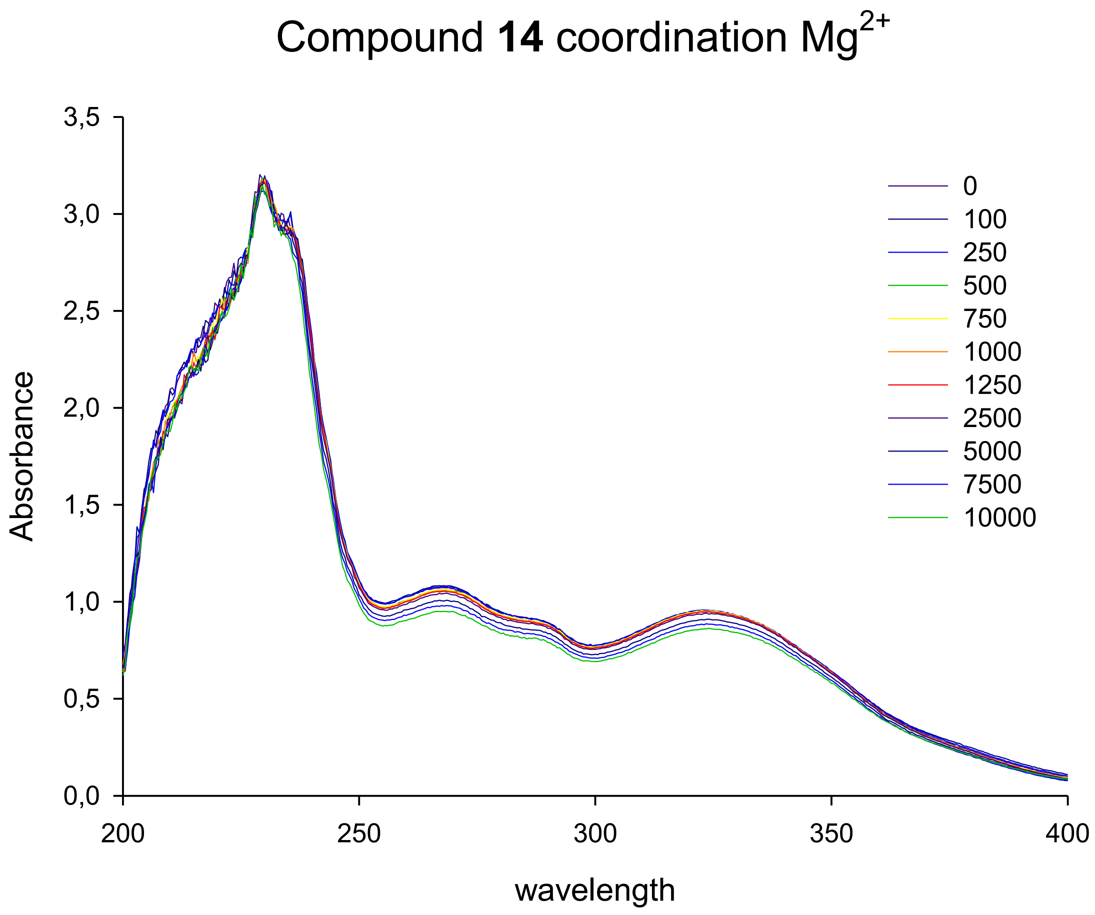
Compound 14 coordination Mg^{2+}
Absorbance
wavelength
3,5
3,0
2,5
2,0
1,5
1,0
0,5
0,0
200
250
300
350
400
0
100
250
500
750
1000
1250
2500
5000
7500
10000

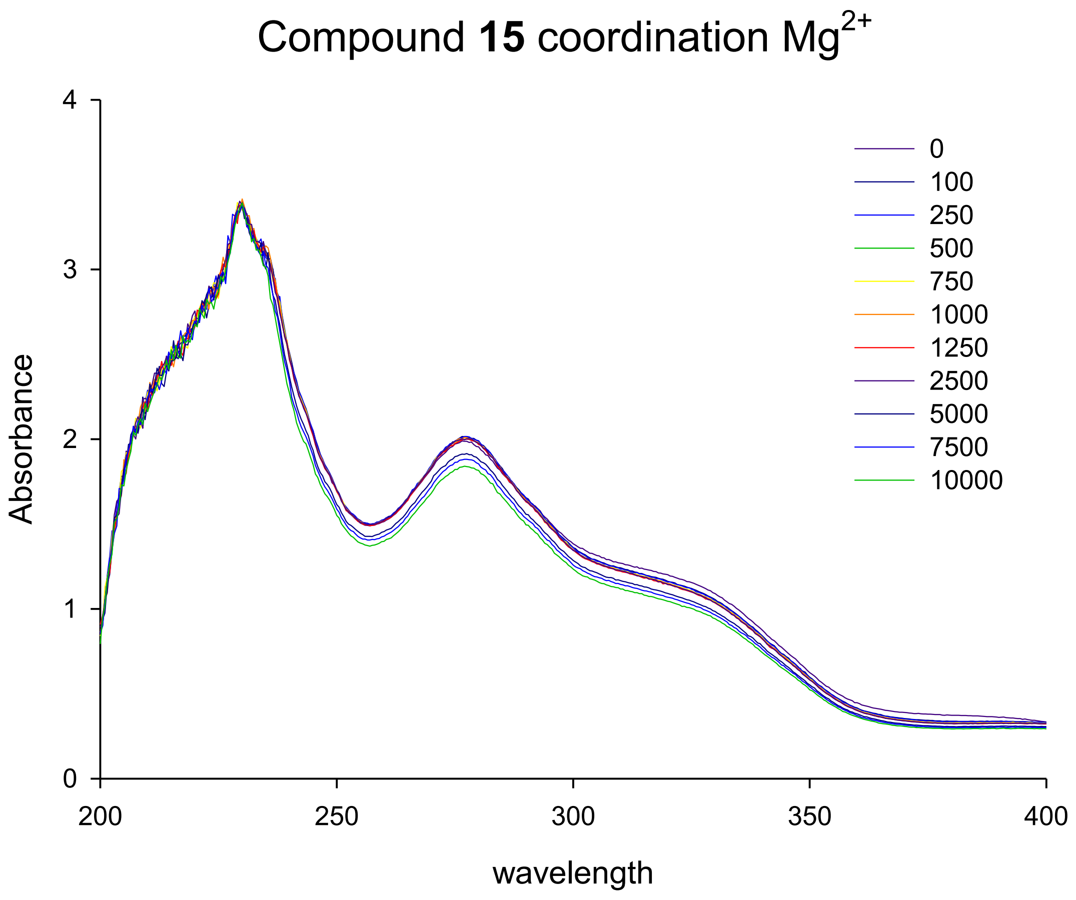
Compound 15 coordination Mg^{2+}
Absorbance
wavelength
4
3
2
1
0
200
250
300
350
400
0
100
250
500
750
1000
1250
2500
5000
7500
10000

Compound **16** coordination Mg^{2+}

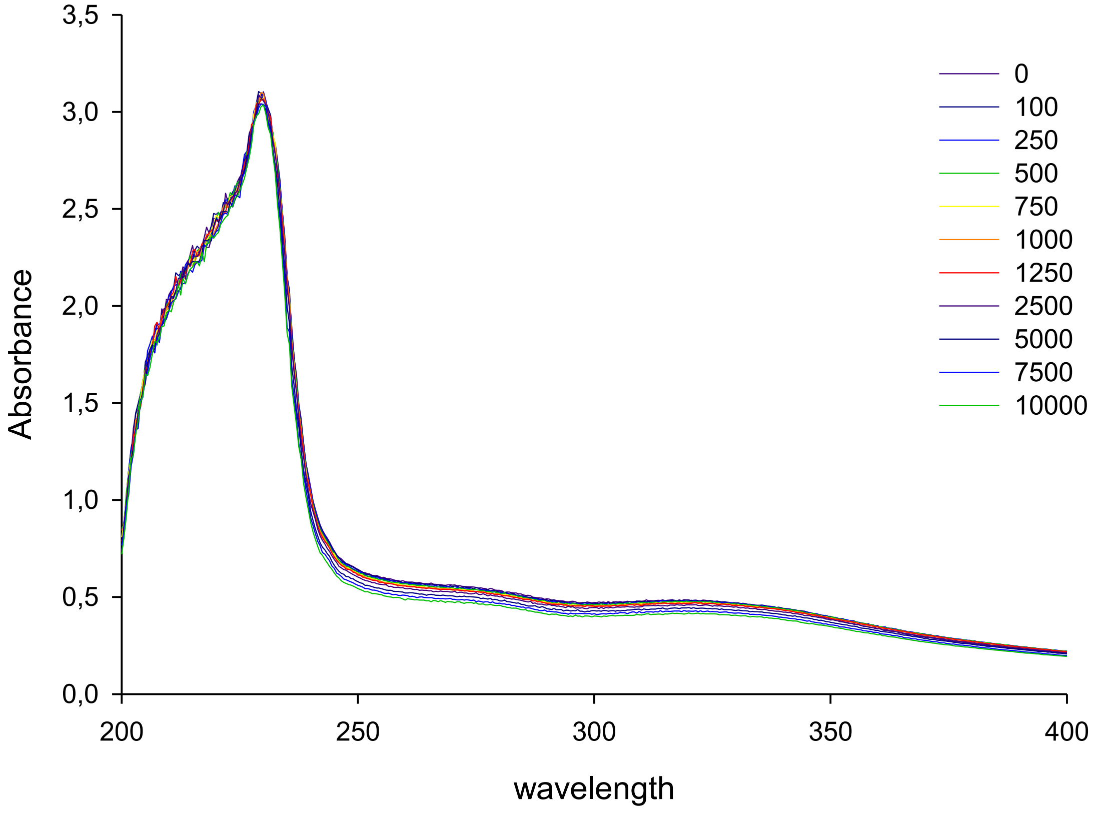

Compound **17** coordination Mg^{2+}

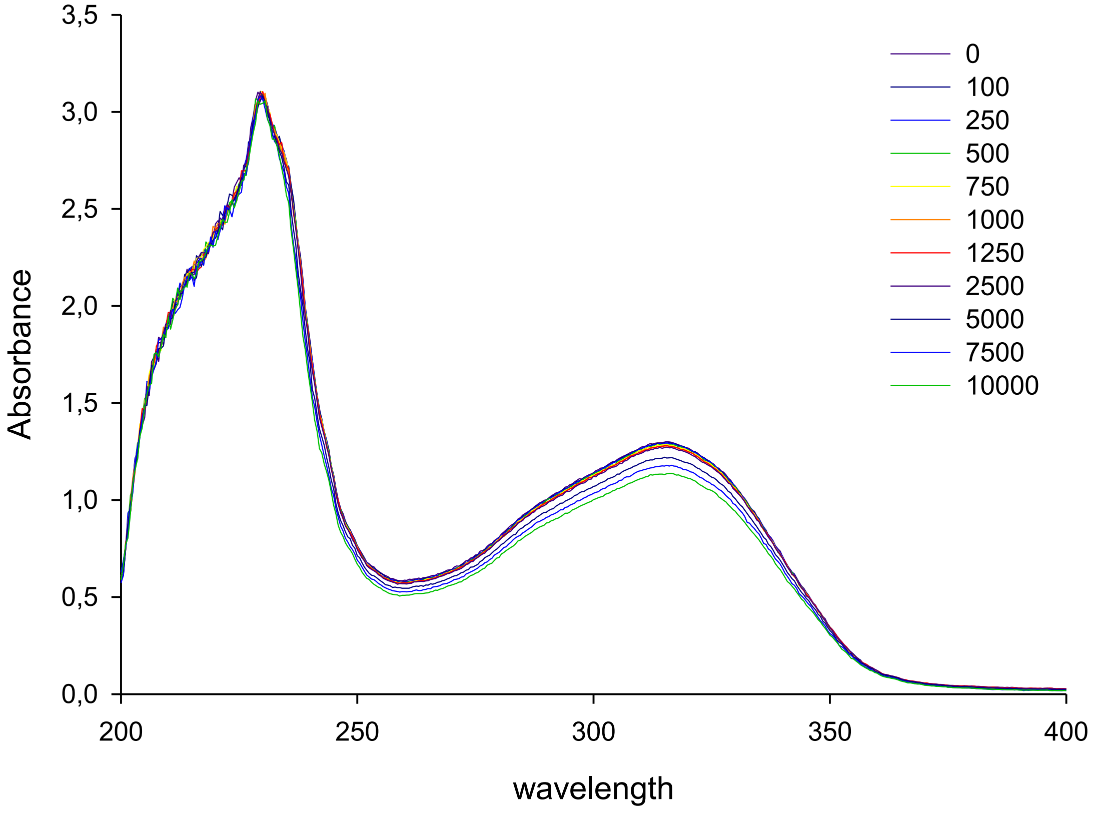

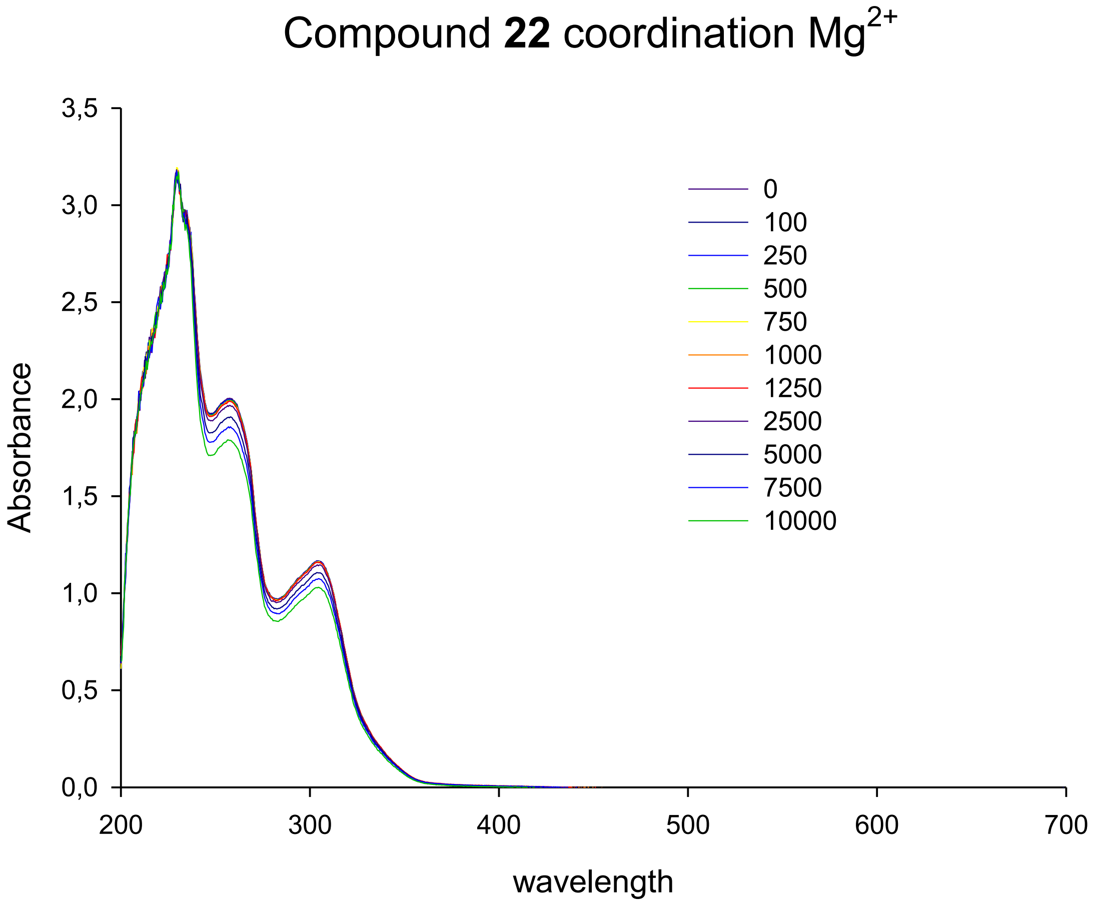
Compound 22 coordination Mg^{2+}
Absorbance
wavelength
0,0
0,5
1,0
1,5
2,0
2,5
3,0
3,5
200
300
400
500
600
700
0
100
250
500
750
1000
1250
2500
5000
7500
10000
